# Extract from *Aronia melanocarpa* L. Berries Prevents Cadmium-Induced Oxidative Stress in the Liver: A Study in A Rat Model of Low-Level and Moderate Lifetime Human Exposure to this Toxic Metal

**DOI:** 10.3390/nu11010021

**Published:** 2018-12-21

**Authors:** Magdalena Mężyńska, Małgorzata M. Brzóska, Joanna Rogalska, Barbara Piłat-Marcinkiewicz

**Affiliations:** 1 Department of Toxicology, Medical University of Bialystok, Adama Mickiewicza 2C street, 15-222 Bialystok, Poland; joanna.rogalska@umb.edu.pl; 2 Department of Histology and Embryology, Medical University of Bialystok, Jerzego Waszyngtona 13 street, 15-269 Bialystok, Poland; barbara.pilat-marcinkiewicz@umb.edu.pl

**Keywords:** *Aronia melanocarpa* berries, cadmium, liver, liver enzymes markers, morphology, oxidative/antioxidative balance, oxidative stress, protection

## Abstract

The study investigated, in a rat model of low-level and moderate environmental exposure to cadmium (Cd; 1 or 5 mg Cd/kg diet, respectively, for 3 to 24 months), whether the co-administration of 0.1% extract from *Aronia melanocarpa* L. berries (AE) may protect against oxidative stress in the liver and in this way mediate this organ status. The intoxication with Cd, dose- and duration-dependently, weakened the enzymatic antioxidative barrier, decreased the concentrations of reduced glutathione and total thiol groups, and increased the concentrations of oxidized glutathione, hydrogen peroxide, xanthine oxidase, and myeloperoxidase in this organ. These resulted in a decrease in the total antioxidative status, increase in the total oxidative status and development of oxidative stress (increased oxidative stress index and malondialdehyde concentration) and histopathological changes in the liver. The administration of AE at both levels of Cd treatment significantly improved the enzymatic and nonenzymatic antioxidative barrier, decreased pro-oxidant concentration, and protected from the development of oxidative stress in the liver and changes in its morphology, as well as normalized the serum activities of liver enzymes markers. In conclusion, consumption of aronia products may prevent Cd-induced destroying the oxidative/antioxidative balance and development of oxidative stress in the liver protecting against this organ damage.

## 1. Introduction

Nowadays, the growing interest of the scientific community, including nutritionists, has been focused on the possibility of using various compounds naturally occurring in plants, not only in the prevention and treatment of civilization diseases, but also in the protection against the unfavorable outcomes of exposure to environmental pollutants, including toxic heavy metals such as cadmium (Cd) [1,2,3,4,5,6]. Especially interesting in this regard are fruits, vegetables, and herbs, which are a rich source of substances characterized by antioxidative properties and could be included in the human diet [1,2,3,4,5,6]. 

Cd belongs to the most toxic pollutants in industrialized and developing countries and forecasts show that exposure to this xenobiotic will increase [6,7,8]. For the general population food is the main source of intoxication with this element [6,7,8]. Moreover, a significant source of chronic exposure to this metal is also habitual tobacco smoking [9,10]. Environmental exposure to Cd at levels nowadays occurring in numerous countries has been reported to create a risk of damage to the kidneys [2,11], skeleton [2,12], and cardiovascular system [2,13], as well as the deterioration of sight and hearing [14,15] and development of cancer [2,16,17]. Moreover, some data show that even low-level exposure to this element may also lead to the injury to the liver [2,18,19]. 

The liver, apart from the kidneys, is the main place of Cd accumulation in the organism and the critical target organ for its toxicity [1]. This organ, due to its role in the biotransformation (detoxification or metabolic activation), storage of numerous xenobiotics that enter the body from various sources (mainly contaminants of food and drinking water, medicines, and ethyl alcohol), and some endogenous substances, is especially prone to damage by various noxious compounds and poisonous products of their biotransformation, including free radicals (FR) [1]. Because the liver plays a key role in the proper functioning of the organism, its dysfunction has numerous negative outcomes [20,21,22]. Thus, it is very important to find effective agents allowing to improve the function of this organ and protect it from damage by various xenobiotics, including Cd which is accumulated in the hepatic tissue during a lifetime. 

Oxidative stress has been recognized as one of the main mechanisms of the toxic action of Cd, including its damaging impact on the liver [1,2,23,24,25,26]. Although this heavy metal is not capable of generating FR and reactive oxygen species (ROS) directly, it can mediate their production indirectly by weakening the enzymatic and nonenzymatic antioxidative barrier, damage to the mitochondria, and induction of the activity of oxidases [1,2,23,24,25,26]. Due to the pro-oxidative properties of Cd, there has been growing interest of the scientific community in the possibility of using natural agents with antioxidative potential in order to prevent from the unfavorable effects of exposure to this xenobiotic and for treatment of them [1,2,3,6]. 

Based on the recent findings of our research team [26,27,28,29,30] and some data by other authors [31], it seems that a very promising natural agent in the protection from harmful effects of exposure to Cd exists in the berries of *Aronia melanocarpa* L. (*A. melanocarpa*, (*Michx.*) Elliott, *Rosaceae*). Aronia berries (chokeberries) are a rich source of polyphenolic compounds, which are one of the most powerful and widespread groups of natural antioxidants [4,5,32]. Owing to the large number of hydroxyl groups (-OH groups), these compounds are capable of chelating metal ions, including Cd ions (Cd^2+^) [32,33,34]. Apart from polyphenols, chokeberry is also abundant in other ingredients characterized by an ability to detoxify FR and ROS, such as vitamins, essential bioelements, carotenoids, phytosterols, and triterpenes [4].

Our studies, conducted in a female rat model of low-level and moderate (1 and 5 mg Cd/kg diet, respectively, for up to 24 months) lifetime human exposure to Cd, revealed that a 0.1% extract from *A*. *melanocarpa* berries (AE) decreased the body burden of Cd (Table S1), including its accumulation in the liver (Figure S1 and Table S1) and kidneys [28], and offered significant protection against this heavy metal-induced damage to the skeleton [26,29,30]. The extract improved the oxidative/antioxidative balance of the serum and bone tissue at the exposure to Cd [26] and prevented against disturbances in the metabolism of zinc (Zn) and copper (Cu), including the liver status of these bioelements [27]. Moreover, the consumption of aronia anthocyanins under exposure to Cd was shown to diminish the storage of this toxic metal in the liver and kidneys and to decrease the serum activities of enzymatic markers of liver injury [31].

Taking into account strong antioxidative potential of AE [4,5,26] and some of the above findings of our research on the protective impact of the extract under exposure to Cd [26,27,28], we have hypothesized that administration of the chokeberry extract under chronic low-level and moderate exposure to this heavy metal may also improve the oxidative/antioxidative status of the liver and in this way prevent from the development of oxidative stress in this organ and its damage. The aim of the present study was to investigate this hypothesis. For this reason numerous markers of oxidative and antioxidative status and the level of oxidative stress were determined in the liver tissue in the experimental model of exposure to Cd (1 and 5 mg Cd/kg diet for up to 24 months) and/or the administration of 0.1% AE that we used previously [26,27,28,29,30]. Moreover, the morphology of this organ and the activities of liver enzymes markers in the serum [35] were evaluated to recognize whether the possible beneficial impact of AE on the oxidative/antioxidative balance in the liver under exposure to Cd may mediate the morphological structure and function of this organ. To the best of our knowledge such investigation has not been conducted until now. 

## 2. Materials and Methods

### 2.1. Ethics Statement

The study gained approval of the Local Ethics Committee for Animal Experiments in Bialystok (Poland; approval numbers 60/2009 on 21 September 2009 and 34/2015 on 25 March 2015). All procedures were performed according to the ethical principles and institutional guidelines, as well as the International Guide for the Use of Animals in Biomedical Research. 

### 2.2. Cd Diets

Diets containing 1 and 5 mg Cd/kg (Labofeed H and Labofeed B diets) were prepared by the addition of cadmium chloride (CdCl_2_ × 2½ H_2_O; POCh; Gliwice, Poland) into the components of the standard Labofeed H diet (breeding diet ensuring the proper growth and development of young animals) and Labofeed B diet (maintenance diet) at the stage of their production by Label Food “Morawski’’ Kcynia. Cd concentration in the fodder was requantified in our laboratory (using the atomic absorption spectrometry method) and it amounted to 1.09 ± 0.13 mg/kg in the 1 mg Cd/kg diet (mean ± standard deviation—SD) and 4.92 ± 0.53 mg/kg in the 5 mg Cd/kg diet [28]. This metal concentration determined in the standard Labofeed diets was 0.0584 ± 0.0049 mg/kg [28].

### 2.3. A. Melanocarpa Extract

A certified (Certificate KJ 4/2010) powdered AE was provided by Adamed Consumer Healthcare (Tuszyn, Poland). According to the manufacturer’s declaration the content of polyphenols in the extract reached 65.74%, therein 18.65% of anthocyanins. The polyphenolic profile of the extract is presented in Table S2 [28,29]. The extract contained also other components such as carotenoids, pectins, sugar, sugar alcohols (sorbitol and parasorboside), triterpenes, and phytosterols, as well as minerals and vitamins [4]. 

The 0.1% aqueous solution of AE was prepared daily by dissolving 1 g of the powdered extract in 1 L of redistilled water. The total concentration of polyphenols in the 0.1% AE was 0.612 ± 0.003 mg/mL (mean ± standard error—SE) [28,29] and the concentration of Cd did not exceed 0.05 µg/L [28]. 

### 2.4. Animal Model

The investigation was carried out on 192 young (3–4 weeks old) female Wistar rats (Crl: WI (Han)) obtained from the certified Laboratory Animal House in Brwinów (Poland). Throughout the experiment all animals were kept in stainless-steel cages in controlled standard conditions (12-h light/dark cycle, temperature 22 ± 2 °C, and relative humidity 50 ± 10%) and had free access to food (the Labofeed H diet throughout the first 3 months of the study and next the Labofeed B diet without and with Cd addition) and drinking water (redistilled water or the 0.1% AE). 

After 5 days of acclimatization, the rats were randomly allocated to 6 experimental groups of 32 animals each. One group received 0.1% AE as the only drinking fluid (AE group), two groups were exposed to Cd via diet at the concentration of 1 and 5 mg Cd/kg (Cd_1_ group and Cd_5_ group, respectively), while the next two groups received the diet containing Cd (1 or 5 mg Cd/kg) and 0.1% AE simultaneously (Cd_1_ + AE group and Cd_5_ + AE group) for 3, 10, 17, and 24 months. The last group, maintained on redistilled water (containing < 0.05 µg Cd/L) and standard Labofeed diet (without the addition of Cd), served as a control. The experimental model has been described in details in our previous reports [26,27,28,29,30].

The daily consumption of the extract by rats reached from 63.1 to 159.1 mg/kg b.w., while the intake of polyphenols ranged from 41.5 to 104.6 mg/kg b.w. (Table S3) and was higher than the recommended daily intake of these compounds [28]. The daily intake of Cd within the 24-month exposure to the 1 and 5 mg Cd/kg diet ranged from 37.50 to 84.88 μg/kg b.w. and from 196.69 to 404.76 μg/kg b.w., respectively (Table S3) [28]. The mean intake of the extract and Cd throughout the study did not differ depending on whether they were administered in combination or separately (Table S3) [28]. Cd concentration in the blood and urine (markers of exposure to this xenobiotic) of the rats treated with the 1 mg Cd/kg diet (0.103–0.324 µg/L and 0.085–0.354 µg/g creatinine, respectively) and 5 mg Cd/kg diet (0.584–1.332 µg/L and 0.284–0.820 µg/g creatinine, respectively), alone or together with AE (Table S1) [28] was within the range of this metal concentrations noted in the general population in industrialized countries [11,12,13,14,15,16,17,18,19]. These confirm that the used experimental model corresponds well with low-level and moderate human environmental exposure to this xenobiotic [2,11,12,13,14,15,16,17,18,19,28]. 

Throughout the whole investigation, no statistically significant differences in the consumption of food and drinking water, or body weight gain were observed among the experimental groups [28]. Although during the study there were no symptoms of abnormalities in health status; 3 cases of unprompted deaths were noted between the 17th and 24th month of the experiment in the AE, Cd_1_, and Cd_5_ groups (one case in each group). 

At termination, after overnight fasting, the rats were subjected to barbiturate anesthesia (Morbital; Biowet, Pulawy, Poland; 30 mg/kg b.w., intraperitoneally). The whole blood was collected by cardiac puncture with and without anticoagulant (heparin; Biochemie GmbH, Kundl, Austria) and various organs and tissues, including the liver, were dissected after macroscopic examination. A portion of the whole blood taken without anticoagulant was centrifuged after coagulation and the serum was separated. Immediately after collection, the liver was rinsed with a cold physiological saline and next it was gently dried on filter paper and weighted (OHAUS, Nanikon, Switzerland; accuracy to 0.0001 g). The left lobe of the liver, dissected from all animals, was separated for morphological examination. The remaining part of the organ and the serum were stored frozen at −80 °C for further studies. 

### 2.5. Determination of the Oxidative/Antioxidative Status of the Liver

#### 2.5.1. Preparation of Homogenates of the Liver Tissue 

Preweighted slices of the liver tissue were homogenized in a cold potassium phosphate buffer (50 mM, pH = 7.4; prepared by mixing 1 M potassium dihydrogen phosphate, 1 M dipotassium hydrogen phosphate (POCh), and distilled water) with the addition of butyl-hydroxytoluene as an antioxidant (Sigma-Aldrich Gmbh, Steinheim, Germany) by using a high-performance homogenizer (Ultra-Turrax T25; IKA, Staufen, Germany) to obtain 10% homogenates (1 mg of the liver tissue was used per each 9 mL of the potassium phosphate buffer). All homogenates were divided into two portions: one of them was centrifuged (MPW-350R centrifugator, Medical Instruments, Warsaw, Poland) at 700× *g* for 20 min at 4 °C, whereas the second was centrifuged at 20000× *g* for 30 min at 4 °C [36]. After the centrifugation the aliquots were separated and stored frozen at −80 °C until all measurements were performed. 

#### 2.5.2. Determination of the Parameters of the Oxidative/Antioxidative Status and the Level of Oxidative Stress

In order to evaluate the oxidative/antioxidative status of the liver, numerous biomarkers of enzymatic (superoxide dismutase—SOD, catalase—CAT, glutathione peroxidase—GPx, glutathione reductase—GR, and glutathione S-transferase—GST) and nonenzymatic (reduced glutathione—GSH, thioredoxin—Trx, and total thiol groups—TSH) antioxidative barriers were determined. Moreover, the concentration of oxidized glutathione (GSSG) was measured in order to evaluate the ratio of GSH/GSSG. The total antioxidative status (TAS) and total oxidative status (TOS) of the liver were assayed, and the oxidative stress index (OSI = TOS/TAS) and the concentration of malondialdehyde (MDA; a marker of lipid peroxidation), as indicators of the intensity of oxidative stress, were evaluated. The concentrations of hydrogen peroxide (H_2_O_2_), xanthine oxidase (XOD), and myeloperoxidase (MPO) were evaluated as well. SOD, GPx, and GR were quantified in the aliquots separated after centrifugation at 20000× *g*, whereas other parameters were assayed in the aliquots separated after centrifugation at 700× *g* [36]. 

The activity of total SOD (Cu, Zn-SOD, and Mn-SOD) was measured by using the commercial kit purchased from Cayman Chemical Company (Ann Arbor, MI, USA). The assay utilizes a tetrazolium salt for detection of superoxide radicals (O_2_^**·**-^) generated by XOD and hypoxantine. Determination of CAT activity was made according to the spectrophotometric method by Aebi [37] based on the measurement of the amount of H_2_O_2_ (CHEMPUR, Piekary Śląskie, Poland) degraded by CAT. The vanishing of H_2_O_2_ was tracked spectrophotometrically at 240 nm. The activity of GPx was assayed by using the Bioxytech GPx-340 kit by OxisResearch (Burlingame, CA, USA). In the assay, GPx is used to catalyze the oxidation of GSH to GSSG initiated by the addition of tertbutyl hydroperoxide. Next, GSSG is reduced by GR and during this process the reduced form of nicotinamide adenine dinucleotide phosphate (NADPH) is transformed into its oxidized form—NADP^+^, which is accompanied by a decrease in the absorbance at 340 nm. The rate of the decrease in the absorbance is directly proportional to the activity of GPx in the sample. The activity of GR was estimated with the use of the Bioxytech GR-340 kit by OxisResearch (Foster City, CA, USA). The method leans on the oxidation of NADPH to NADP^+^ in the process of GSSG reduction catalyzed by limited concentration of GR. One molecule of NADPH is utilized for the reduction of one molecule of GSSG. The depletion of GSSG is determined by the measurement of NADPH consumption and demonstrated as a decrease in the absorbance at 340 nm. 

The concentrations of GSH and GSSG were estimated colorimetrically using the Glutathione Assay Kit by Cayman Chemical Company. GSH was determined based on the absorbance (at 405–412 nm) of 5-thio-2-nitrobenzoic acid (TNT), as the product of the reaction between GSH and 5,5’-dithio-bis-2-nitrobenzoic acid (DTNB). Quantification of GSSG was accomplished by first derivatizing GSH with 2-vinylpyridine. The liver concentration of TSH was determined with the method by Ellman [38] based on the colorimetric measurement of TNT formed in the reaction between -SH groups in the sample and DTNB at 412 nm [38].

The concentration of H_2_O_2_ was assayed using the Bioxytech H_2_O_2_-560 kit by OxisResearch (Portland, OR, USA) based on this compound-induced oxidation of ferrous iron (Fe^2+^) to ferric ion (Fe^3+^). The liver concentrations of GST, Trx, XOD, and MPO were determined with the use of specific Rat(GST), Rat(Trx), Rat(XOD), and Rat(MPO) double-antibody sandwich enzyme-linked immunosorbent assay (ELISA) kits by SunRed (Shanghai, China).

The liver TAS was assayed using the ImAnOx (TAS) ELISA kit by Immundiagnostik AG (Bensheim, Germany). The method is based on the reaction of elimination of added H_2_O_2_ by antioxidants present in an investigated sample. The residual H_2_O_2_ generates products which absorb at 450 nm. The value of TOS was determined with the PerOx (TOS) ELISA kit by Immundiagnostik AG based on the measurement of total lipid peroxides present in the investigated sample in the reaction with peroxidase at 450 nm. The concentration of MDA in the liver was evaluated by using Bioxytech LPO-586 kit by OxisResearch (Burlingame, CA, USA). The assay is based on the reaction of a chromogenic reagent, N-methyl-2-phenylindole, with MDA at 45 °C resulting in the formation of a stable chromophore with maximal absorbance at 586 nm. 

All analyses performed with the use of commercial kits were done strictly in accordance to the manufacturers’ instructions. The quantification of investigated parameters was performed with the use of ELISA universal microplate reader (BIO-TEK Instruments Inc, ELX_800_, Winooski, USA) and U-3010 spectrophotometer by Hitachi (Tokyo, Japan). The quality control of all measurements (Supplementary Material—Analytical Quality of Measurements) confirmed the reliability of the obtained results. All parameters, except for ALT and AST, were adjusted for protein concentration (assayed with the use of BioMaxima kit, Lublin, Poland). 

### 2.6. Estimation of the Liver Morphology and the Activities of Liver Enzymes Markers in the Serum 

Slices of the left lobe of the liver were fixed in Bouin’s solution (prepared by mixing picric acid (Sigma-Aldrich Gmbh), formalin (CHEMPUR), and acetic acid (CHEMPUR)) for 24 h, dehydrated in different concentrations of ethyl alcohol (POCh), cleared with xylene (POCh), and embedded in paraffin (CHEMPUR). Sections of the liver tissue (4–5 µm thick) were prepared by using rotary microtone (Leica, Nussloch, Germany). Next, the sections were stained with hematoxylin (“AQUA-MED” ZPAM-KOLASA, Lódź, Poland) and eosin (Analab, Warsaw, Poland; H + E staying), and examined under a light microscope (Olympus, Tokyo, Japan) for pathological changes. A semistatistical evaluation of the changes in the liver morphology was performed. The following criteria were used to scoring the presence or absence of the changes: +++, a change was found in most of the lobes, in all animals of a group; ++, a change was found in some lobes, in all animals of a group; +, a change was found in some lobes in a majority of animals of a group (occurred in 5–7 animals); ±, a change was sporadic in a group (occurred in 1 or 2 animals); and -, a change was absent in all animals of a group; ^a^ a change was present in single cells in a group; ^b^ a change was found in some cells in lobes; ^c^ a change occurred in a majority of cells in lobes; and ^d^ a change was found in almost all cells in lobes.

The activities of the main liver enzymes markers such as alanine aminotransferase (ALT) and *aspartate transaminase* (AST) in the serum were measured with the use of diagnostic kits by BioMaxima (Lublin, Poland).

### 2.7. Statistical Analysis

Statistical analysis of the results was performed using the Statistica 12 package (StatSoft, Tulsa, USA). A one-way analysis of variance (ANOVA) with Duncan’s multiple range post hoc test was carried out for comparisons between individual groups and to determine if the differences between these groups were statistically significant (*p* < 0.05). At the beginning, ANOVA was performed to determine whether there were statistically significant differences among the six experimental groups, and when the differences occurred, Duncan’s multiple range post hoc test was performed to determine which two means differed (*p* < 0.05). In the figures, statistically significant differences in relation to the control group, the respective group receiving Cd alone (Cd_1_ + AE vs. Cd_1_ and Cd_5_ + AE vs. Cd_5_), the group receiving AE alone (Cd_1_ vs. AE, Cd_1_ + AE vs. AE, Cd_5_ vs. AE, and Cd_5_ + AE vs. AE), and the respective group exposed to the 1 mg Cd/kg diet alone or with AE (Cd_5_ vs. Cd_1_ and Cd_5_ + AE vs. Cd_1_ + AE) are marked. In the case when the post hoc analysis revealed any influence of the co-administration of Cd and AE on the investigated parameter, the possible interactive and independent effects of Cd and AE action were evaluated with the use of a two-way analysis of variance (ANOVA/MANOVA, test *F*). *F* values having *p* < 0.05 were taken to indicate a statistically significant effect. Moreover, in order to estimate the dependence between the liver burden of Cd and the extent of destroying the oxidative/antioxidative balance in this organ, the relationships between the main indices of oxidative stress measured in this study (TOS, OSI, MDA, and H_2_O_2_), as well as enzymatic markers of the liver function (ALT and AST) and the concentration of Cd in the liver (Table S1), presented in our previous reports from studies in this experimental model [28], were evaluated with the use of Spearman rank correlation analysis. Correlations were considered statistically significant at *p* < 0.05.

## 3. Results

### 3.1. Effect of AE and/or Cd on the Enzymatic Antioxidative Barrier of the Liver

**Figure 1:**
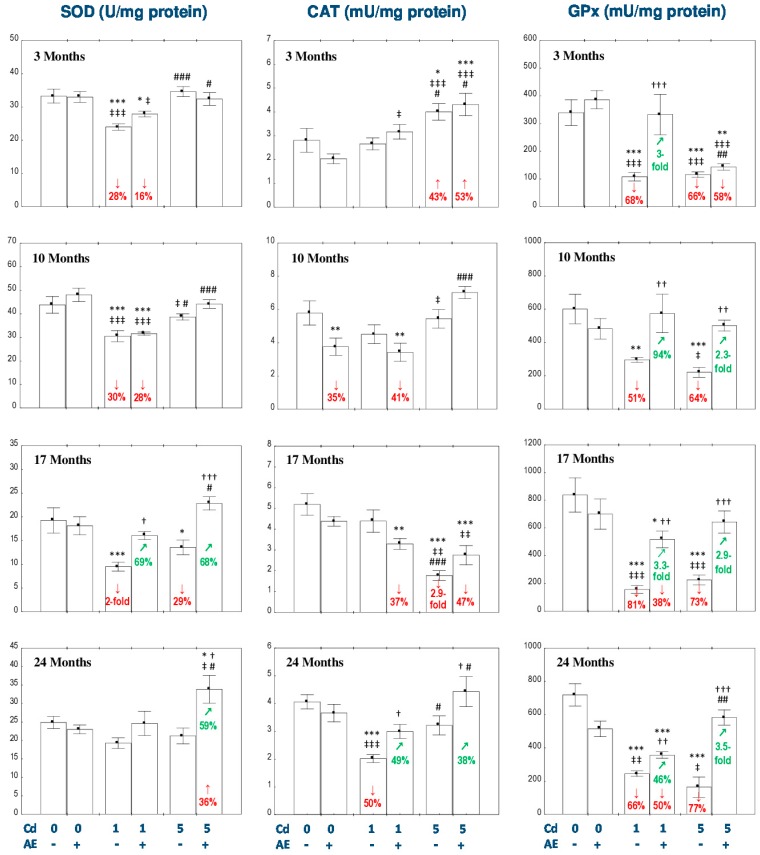
The effect of the extract from the berries of *Aronia melanocarpa* L. (AE) on the activities of superoxide dismutase (SOD), catalase (CAT), and glutathione peroxidase (GPx) in the liver of rats exposed to cadmium (Cd). The rats received Cd in the diet at the concentration of 0, 1, and 5 mg Cd/kg and/or 0.1% aqueous AE (+) or not (-). Data are presented as mean ± SE for 8 rats, except for seven animals in the AE, Cd_1_, and Cd_5_ group after 24 months. Statistically significant differences (ANOVA, Duncan’s multiple range test): * p < 0.05, ** *p* < 0.01, *** *p* < 0.001 vs. control group; ^†^ *p* < 0.05, ^††^ *p* < 0.01, ^†††^ *p* < 0.001 vs. respective group intoxicated with Cd alone; ^‡^ *p* < 0.05, ^‡‡^ *p* < 0.01, ^‡‡‡^ *p* < 0.001 vs. group receiving AE alone; ^#^ *p* < 0.05, ^##^ *p* < 0.01, ^###^ *p* < 0.001 vs. respective group receiving the 1 mg Cd/kg diet (alone or with AE). Numerical values in bars disclose the percentage changes or factors of changes in comparison to the control group (↓, decrease; ↑, increase) or the respective group receiving Cd alone (↗, increase).

**Figure 2:**
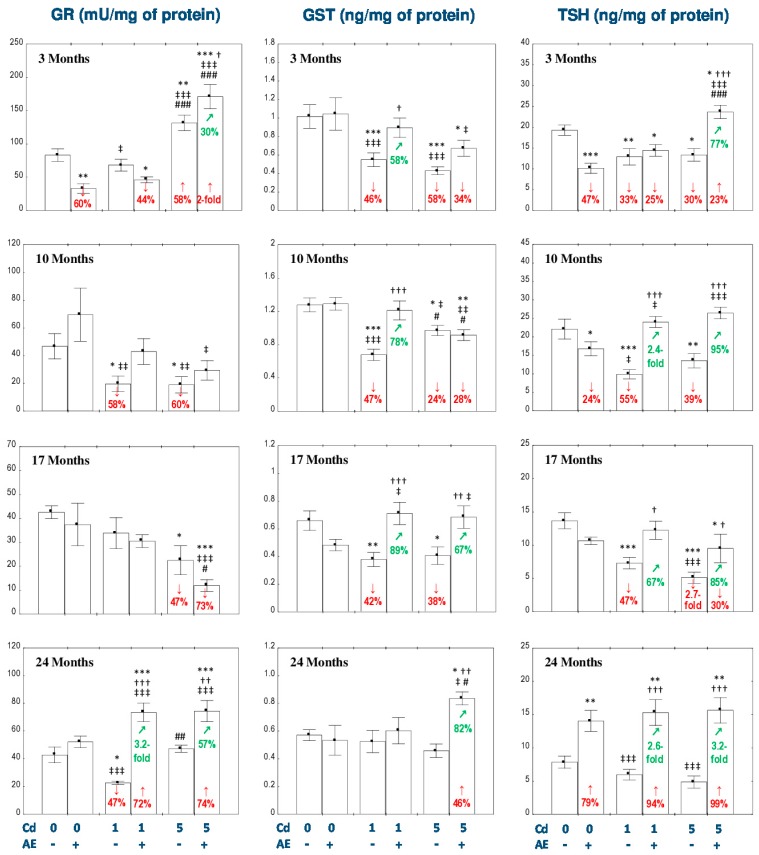
The effect of the extract from the berries of *Aronia melanocarpa* L. (AE) on the activity of glutathione reductase (GR) and the concentrations of glutathione S-transferase (GST) and total thiol groups (TSH) in the liver of rats exposed to cadmium (Cd). The rats received Cd in the diet at the concentration of 0, 1, and 5 mg Cd/kg and/or 0.1% aqueous AE (+) or not (-). Data are presented as mean ± SE for eight rats, except for seven animals in the AE, Cd_1_, and Cd_5_ group after 24 months. Statistically significant differences (ANOVA, Duncan’s multiple range test): * p < 0.05, ** *p* < 0.01, *** *p* < 0.001 vs. control group; ^†^ *p* < 0.05, ^††^ *p* < 0.01, ^†††^ *p* < 0.001 vs. respective group intoxicated with Cd alone; ^‡^ *p* < 0.05, ^‡‡^ *p* < 0.01, ^‡‡‡^ *p* < 0.001 vs. group receiving AE alone; ^#^ *p* < 0.05, ^##^ *p* < 0.01, ^###^ *p* < 0.001 vs. respective group receiving the 1 mg Cd/kg diet (alone or with AE). Numerical values in bars disclose the percentage changes or factors of changes in comparison to the control group (↓, decrease; ↑, increase) or the respective group receiving Cd alone (↗, increase).

The administration of AE alone for up to 24 months had no impact on the activities of SOD, CAT, GPx, and GR and the concentration of GST in the liver (Figure 1 and Figure 2), except for a decrease in the activities of GR after 3 months (Figure 2) and CAT after 10 months of the study (Figure 1). The consumption of AE alone also decreased the liver concentration of TSH after 3 and 10 months, but after 24 months of the experiment the value of this parameter was higher compared to the control group (Figure 2). 

In the rats receiving the diet containing 1 mg Cd/kg, the activity of SOD in the liver decreased (by 28–100%) after 3–17 months, while the activity of CAT was declined (by 50%) only after 24 months (Figure 1). The administration of AE under exposure to the 1 mg Cd/kg diet completely prevented the Cd-induced decrease in the activity of SOD after 17 months and the activity of CAT after 24 months of the investigation. However, the extract intake did not provide any protection regarding other changes in the activity of SOD and declined the activity of CAT after 10 and 17 months to the values lower (by 41% and 37%, respectively) compared to the control group (Figure 1). In the rats intoxicated with the diet containing 5 mg Cd/kg, the activity of SOD in the liver was decreased (by 29%) only after 17 months, whereas the activity of CAT increased (by 43%) after 3 months and decreased (2.9-fold) after 17 months (Figure 1). The consumption of AE completely prevented the 5 mg Cd/kg diet-caused change in the activity of SOD and markedly increased the activity of this enzyme after 24 months compared to the control group and Cd_5_ group (by 36% and 59%, respectively; Figure 1). The co-administration of AE for 3 and 10 months had no impact on the change of CAT activity by the 5 mg Cd/kg diet, while supplementation with the extract for 24 months enhanced the activity of this enzyme, increasing it (by 38%) compared to the Cd_5_ group (Figure 1). The ANOVA/MANOVA analysis revealed that the modifying effect of AE consumption during the exposure to Cd on the activities of SOD and CAT in the liver was the result of independent action of the extract ingredients (*F* = 4.370–6.852, *p* < 0.05) and/or their interaction with this metal (*F* = 4.961–8.478, *p* < 0.05–0.01), especially under the higher exposure to Cd (Table S4). However, the analysis revealed the lack of an independent effect of AE and its interaction with Cd (Table S4) on the activity of SOD in the Cd_1_ + AE group after 17 months, in spite of the total protective impact of the extract administration under the low-level exposure to this xenobiotic, recognized on the basis of the findings of the one-way analysis of variance (Figure 1). 

The intoxication with the 1 mg Cd/kg diet decreased the activities of GPx in the liver at each time point (by 51–81%; Figure 1) and GR after 10 and 24 months (by 58% and 47%, respectively; Figure 2), as well as the concentration of GST after 3–17 months (by 42–47%; Figure 2). The administration of AE to the animals maintained on the diet containing 1 mg Cd/kg provided total protection from the unfavorable impact of Cd on the activity of GPx after 3 and 10 months, whereas after its longer application the protection was only partial (Figure 1). The co-administration of the chokeberry extract under the 1 mg Cd/kg diet decreased (by 44%) the activity of GR in the liver after 3 months, compared to the control group and completely prevented the influence of Cd on the activity of this enzyme after 10 and 24 months. The activity of GR in the Cd_1_ + AE group after 24 months was higher compared to the control group (by 72%) and Cd_1_ group (3.2-fold; Figure 2). Moreover, the use of AE during the low-level intoxication with Cd for 3, 10, and 17 months completely prevented the decrease in the concentration of GST (Figure 2). Like in the case of the treatment with the diet containing 1 mg Cd/kg, the intoxication with the 5 mg Cd/kg diet decreased the activity of GPx in the liver at each time point (by 64–77%; Figure 1) and the activity of GST during the first 17 months (by 24–58%; Figure 2). The activity of GR at this level of exposure to Cd was increased (by 58%) after 3 months and next it decreased reaching after 10 and 17 months values lower (by 60% and 47%, respectively) compared to the control group (Figure 2). The administration of AE to the rats maintained on the diet containing 5 mg Cd/kg for 10–24 months, but not for 3 months, completely prevented this metal-caused decline in the activity of GPx (Figure 1). The consumption of the extract under the moderate exposure to Cd provided entire protection from the decrease in the activity of GR after 10 months, increased its activity after 3 and 24 months to the values higher compared to both control (by 100% and 74%, respectively) and Cd_5_ group (by 30% and 57%, respectively; Figure 2). Moreover, the supplementation with AE totally prevented from the decrease in the concentration of GST caused by the 17-month consumption of the diet containing 5 mg Cd/kg and increased its concentration after 24 months to the value higher compared to the control group and Cd_5_ group (by 46% and 82%, respectively; Figure 2). The consumption of AE did not provide any protection against the change in the activity of GR induced by the 17-month feeding with the 5 mg Cd/kg diet and the concentration of GST after 3 and 10 months (Figure 2). The modifying impact of AE on the activities of GPx and GR and the concentration of GST in the liver of rats exposed to Cd was caused by independent action of the extract (*F* = 6.998–34.18, *p* < 0.05–0.001) and/or its interaction with this toxic metal (*F* = 6.242–30.31, *p* < 0.05–0.001; Table S5). Nevertheless, the ANOVA/MANOVA analysis revealed the lack of an independent effect of AE and/or its interaction with Cd (Table S5) on the activity of GR in the Cd_1_ + AE and Cd_5_ + AE groups after 10 months in spite of the complete protective impact of the extract intake under the exposure to this heavy metal revealed by one-way analysis of variance (Figure 2). 

As can be evident from the data presented in Figure 1 and Figure 2, the impact of Cd alone and the effect of AE co-administration on the enzymatic antioxidative barrier of the liver was, to some extent, dependent on the level of exposure to this toxic metal. It is important to underline that the values of the activities or concentrations of the determined enzymes (SOD, CAT, GR, GPx, and GST) in the Cd_5_ and/or Cd_5_ + AE groups at some time points reached values higher than in the respective groups maintained on the diet containing 1 mg Cd/kg alone or with the extract (Cd_1_ and Cd_1_ + AE groups; Figure 1 and Figure 2).

### 3.2. Effect of AE and/or Cd on the Nonenzymatic Antioxidative Barrier of the Liver

**Figure 3:**
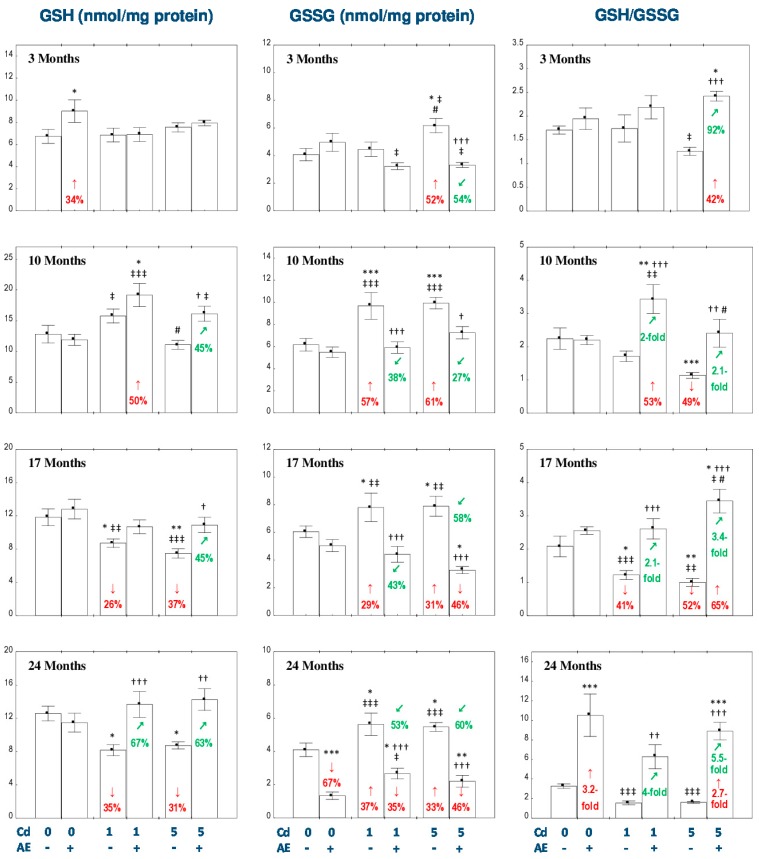
The effect of the extract from the berries of *Aronia melanocarpa* L. (AE) on the concentrations of reduced glutathione (GSH) and oxidized glutathione (GSSG), and the ratio of GSH/GSSG in the liver of rats exposed to cadmium (Cd). The rats received Cd in the diet at the concentration of 0, 1, and 5 mg Cd/kg and/or 0.1% aqueous AE (+) or not (-). Data are presented as mean ± SE for eight rats, except for seven animals in the AE, Cd_1_, and Cd_5_ groups after 24 months. Statistically significant differences (ANOVA, Duncan’s multiple range test): * p < 0.05, ** *p* < 0.01, *** *p* < 0.001 vs. control group; ^†^ *p* < 0.05, ^††^ *p* < 0.01, ^†††^ *p* < 0.001 vs. respective group intoxicated with Cd alone; ^‡^ *p* < 0.05, ^‡‡^ *p* < 0.01, ^‡‡‡^ *p* < 0.001 vs. group receiving AE alone; ^#^ *p* < 0.05 vs. respective group receiving the 1 mg Cd/kg diet (alone or with AE). Numerical values in bars or above the bars disclose the percentage changes or factors of changes in comparison to the control group (↓, decrease; ↑, increase) or the respective group receiving Cd alone (↙, decrease; ↗, increase).

The only changes in the concentrations of GSH and GSSG and the ratio of GSH/GSSG in the liver of rats consuming AE alone were an increase in GSH concentration after 3 months, decrease in GSSG concentration after 24 months, and an enhancement of the GSH/GSSG ratio also after 24 months (Figure 3). 

The exposure to the 1 mg Cd/kg diet resulted in a decrease in the concentration of GSH after 17 and 24 months (by 26% and 35%, respectively) and an increase (by 29–57%) in the concentration of GSSG after 10, 17, and 24 months of the treatment (Figure 3). The ratio of GSH/GSSG in the Cd_1_ group was reduced (by 41%) only after 17 months of the intoxication (Figure 3). The consumption of AE under the low-level treatment with Cd totally prevented this metal-provoked decrease in the concentration of GSH and increased its concentration after 10 months to a value higher (by 50%) than in the control group (Figure 3). The co-administration of the extract and the 1 mg Cd/kg diet completely prevented these metal-caused changes in the concentration of GSSG and the ratio of GSH/GSSG (Figure 3). After 24 months, the concentration of GSSG in the Cd_1_ + AE group was even lower (by 35%) compared to the control group (Figure 3). Moreover, the intake of AE increased the GSH/GSSG ratio compared to the control group (by 53%) and Cd_1_ group (by 100%) after 10 months (Figure 3). Apart from that, the ratio of GSH/GSSG increased (4-fold), compared to the Cd_1_ group, as a result of the extract consumption under the 24-month maintenance on the 1 mg Cd/kg diet; however, it did not differ compared to the control group (Figure 3). The exposure to the 5 mg Cd/kg diet led to a decrease in the concentration of GSH after 17 and 24 months (by 37% and 31%, respectively), increased the concentration of GSSG at each time point (by 31–61%), and decreased the ratio of GSH/GSSG after 10 and 17 months (by 49% and 52%, respectively; Figure 3). The supplementation with AE under the feeding with the 5 mg Cd/kg diet totally prevented against the unfavorable impact of this xenobiotic on the concentrations of GSH and GSSG, and the ratio of GSH/GSSG. Moreover, the values of GSSG in the Cd_5_ + AE group after 17 and 24 months were even lower (by 46%) than in the control group, whereas the GSH/GSSG ratio was higher (from 42% up to 2.7-fold) throughout the study compared to the control group, except for 10 months (Figure 3).

The two-way analysis of variance showed that the impact of AE administration on the concentrations of GSH and GSSG, as well as the ratio of GSH/GSSG was the result of independent action of the extract ingredients (*F* = 4.752–81.91, *p* < 0.05–0.001) and/or their interaction with this metal (*F* = 4.312–15.52, *p* < 0.05–0.001; Table S6). However, the ANOVA/MANOVA analysis revealed the lack of an independent effect of chokeberry extract and its interaction with Cd (Table S6) on GSH concentration in the Cd_1_ + AE group after 10 and 17 months, in spite of the clear impact of AE administration under the exposure to Cd recognized on the basis of the findings of one-way analysis of variance (Figure 3). 

In the Cd_5_ group, the concentration of GSH after 10 months of the experiment was lower (by 30%) than in the Cd_1_ group, while the concentration of GSSG after 3 months was higher (by 39%) than at the low-level exposure (Figure 3). The ratio of GSH/GSSG in the Cd_5_ + AE group was lower (by 30%) after 10 months and higher (by 32%) after 17 months compared to the Cd_1_ + AE group (Figure 3).

The concentration of TSH in the AE group was decreased after 3 and 10 months and increased after 24 months (Figure 2). In the animals maintained on the diets containing 1 and 5 mg Cd/kg from 3 to 17 months, the concentration of TSH in the liver was decreased (from 30% up to 2.7-fold; Figure 2). The administration of AE under the intoxication with Cd completely or partially prevented all changes in the concentration of TSH caused by this toxic metal, except for the decrease in its concentration in the Cd_1_ + AE group after 3 months (Figure 2). Furthermore, the extract markedly increased the concentration of TSH after its 24-month co-administration with Cd (Figure 2). The concentration of TSH in the Cd_5_ + AE group after 3 months and in the Cd_1_ + AE and Cd_5_ + AE groups after 24 months was higher not only compared to the respective Cd group (by 77%, 2.6-fold, and 3.2-fold, respectively), but even compared to the control group (by 23%, 94%, and 99%, respectively; Figure 2). The ANOVA/MANOVA analysis revealed that the beneficial effect of AE consumption on the concentration of TSH during the feeding with the diet containing 1 mg Cd/kg diet was caused by independent action of the extract ingredients after 10 and 24 months (*F* = 5.346, *p* < 0.05 and *F* = 30.21, *p* < 0.001, respectively) and their interaction with this metal after 10 and 17 months of the study (*F* = 26.05, *p* < 0.05 and *F* = 14.45, *p* < 0.001, respectively; Table S6). The influence of AE on the concentration of TSH under the higher intoxication with Cd was the result of the extract ingredients interaction with this heavy metal (*F* = 7.760–47.82, *p* < 0.05–0.01), except for 24 months, where this effect was the result of independent action of chokeberry extract (*F* = 35.90, *p* < 0.001; Table S6).

The concentration of TSH in the Cd_5_ + AE group was higher (by 64%) compared to the Cd_1_ + AE group after 3 months of the study (Figure 2).

The concentration of Trx in the liver of control animals ranged from 0.152 ± 0.009 to 0.236 ± 0.017 ng/mg protein at particular time points and did not change significantly in all experimental groups throughout the study, except for an increase in the Cd_5_ + AE group compared to the control and Cd_5_ group, after 24 months of the investigation (by 59% and 46%, respectively; Table S7). 

### 3.3. Effect of AE and/or Cd on the Concentration of H_2_O_2_ in the Liver

**Figure 4:**
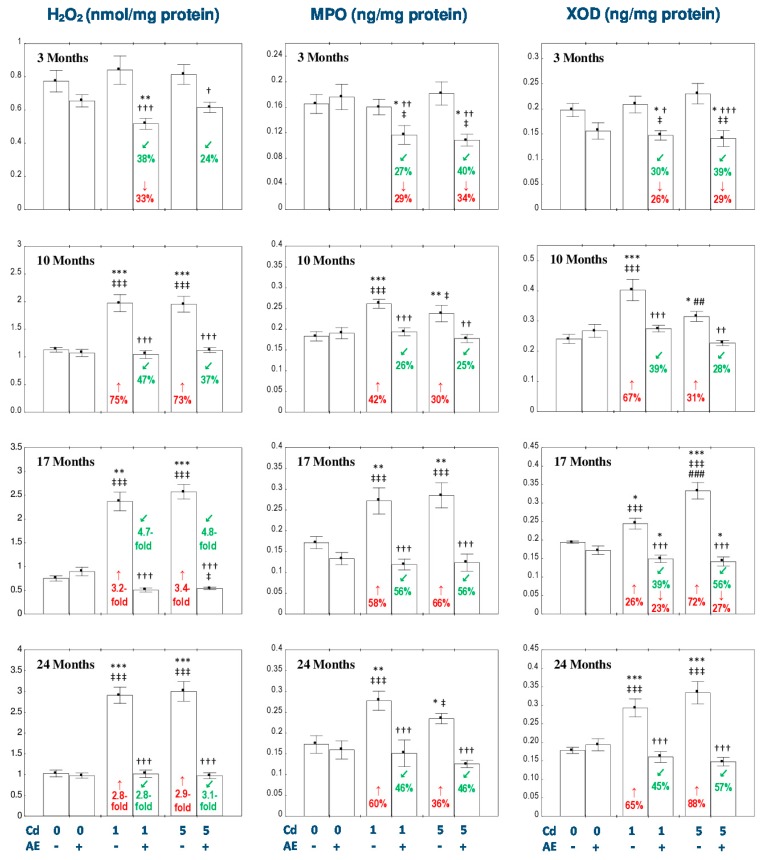
The effect of the extract from the berries of *Aronia melanocarpa* L. (AE) on the concentrations of hydrogen peroxide (H_2_O_2_), myeloperoxidase (MPO), and xanthine oxidase (XOD) in the liver of rats exposed to cadmium (Cd). The rats received Cd in the diet at the concentration of 0, 1, and 5 mg Cd/kg and/or 0.1% aqueous AE (+) or not (-). Data are presented as mean ± SE for eight rats, except for seven animals in the AE, Cd_1_, and Cd_5_ group after 24 months. Statistically significant differences (ANOVA, Duncan’s multiple range test): * p < 0.05, ** *p* < 0.01, *** *p* < 0.001 vs. control group; ^†^ *p* < 0.05, ^††^ *p* < 0.01, ^†††^ *p* < 0.001 vs. respective group intoxicated with Cd alone; ^‡^ *p* < 0.05, ^‡‡^ *p* < 0.01, ^‡‡‡^ *p* < 0.001 vs. group receiving AE alone; ^##^ *p* < 0.01, ^###^ *p* < 0.001 vs. respective group receiving the 1 mg Cd/kg diet (alone or with AE). Numerical values in bars or above the bars disclose the percentage changes or factors of changes in comparison to the control group (↓, decrease; ↑, increase) or the respective group receiving Cd alone (↙, decrease).

The administration of AE alone had no impact on the concentration of H_2_O_2_ in the liver throughout the 24-month experimental period (Figure 4). 

The intoxication with the 1 and 5 mg Cd/kg diet for 10, 17, and 24 months resulted in an increase in the concentration of H_2_O_2_ (from 73% to 3.4-fold), while the co-administration of AE totally prevented against this effect of Cd action (Figure 4). Apart from all that, the consumption of the extract during the 3-month exposure to Cd decreased the concentration of H_2_O_2_ compared to the respective group treated with Cd alone at both levels of exposure and towards the control group at the low-level intoxication (Figure 4). 

According to the results of the ANOVA/MANOVA analysis, the beneficial impact of AE on the concentration of H_2_O_2_ in the liver of rats exposed to Cd was an effect of both independent action of the extract ingredients (*F* = 9.818–103.3, *p* < 0.01–0.001) and/or their interaction with Cd (*F* = 21.02–137.6, *p* < 0.001; Table S8). 

There were no differences in the concentration of H_2_O_2_ in the liver between the respective groups receiving the 1 and 5 mg Cd/kg diet alone or with AE (Figure 4). 

A positive correlation was noted between H_2_O_2_ concentration and Cd concentration in the liver (correlation coefficient – *r* = 0.262, *p* < 0.001).

### 3.4. Effect of AE and/or Cd on the Concentrations of MPO and XOD in the Liver

The concentrations of MPO and XOD in the liver were not influenced by the consumption of AE alone (Figure 4). 

The exposure to the 1 and 5 mg Cd/kg diet increased the concentrations of MPO and XOD in this organ after 10, 17, and 24 months (by 26–88%; Figure 4), while the co-administration of AE completely prevented from this change (Figure 4). Moreover, the concentration of XOD in the Cd_1_ + AE and Cd_5_ + AE groups after 17 months was even lower (by 23% and 27%, respectively) compared to the control group (Figure 4). The consumption of the extract under the 3-month maintaining of the rats on the diet containing 1 and 5 mg Cd/kg decreased the concentrations of MPO and XOD compared to the control group (by 26–34%) and the respective group administered with Cd alone (by 27–40%; Figure 4). 

The ANOVA/MANOVA analysis revealed that the favorable effect of AE consumption on the concentrations of MPO and XOD in the liver during the exposure to Cd was the result of independent action of the extract ingredients (*F* = 5.003–58.18, *p* < 0.05–0.001) and/or their interaction with this heavy metal (*F* = 5.008–36.78, *p* < 0.05–0.001; Table S8). However, the analysis displayed the lack of an independent effect of AE and its interaction with Cd (Table S8) on the concentration of MPO after 3 months in spite of the clear impact of the extract consumption under the low-level exposure to Cd recognized on the basis of the findings of the ANOVA analysis (Figure 4).

There were no differences in the concentrations of MPO and XOD in the liver between the respective groups receiving the 1 and 5 mg Cd/kg diet alone or with AE (Cd_1_ vs. Cd_5_ and Cd_1_ + AE vs. Cd_5_ + AE), except for lower (by 22%) concentration of XOD in the Cd_5_ group compared to the Cd_1_ group after 10 months and higher (by 36%) value of this parameter in the Cd_5_ group after 17 months (Figure 4).

### 3.5. Effect of AE and/or Cd on TAS and TOS and the Level of Oxidative Stress in the Liver 

**Figure 5:**
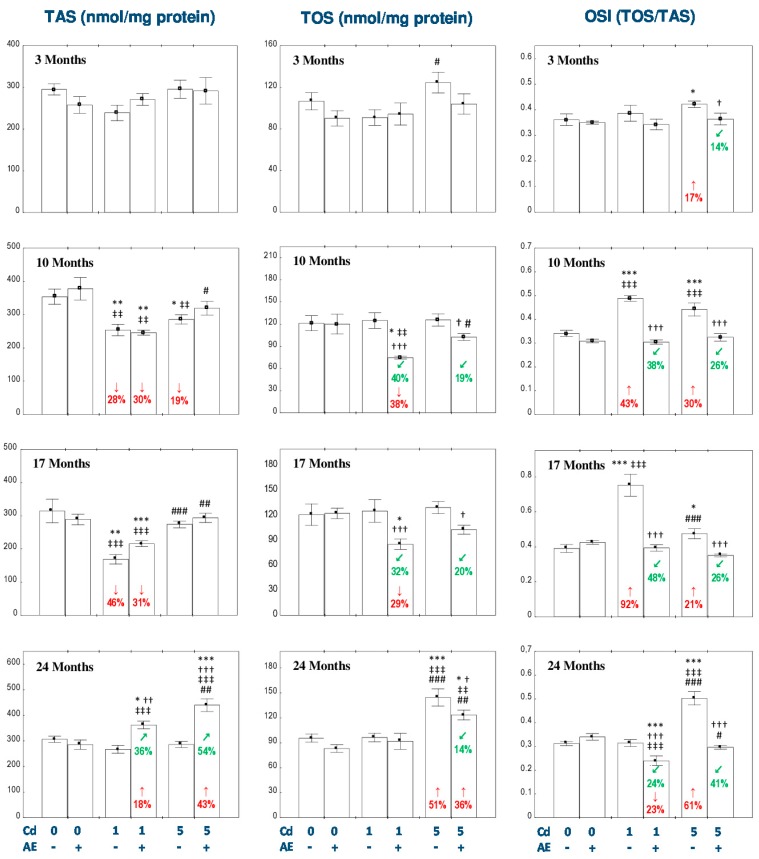
The effect of the extract from the berries of *Aronia melanocarpa* L. (AE) on the total antioxidative status (TAS), total oxidative status (TOS), and the oxidative stress index (OSI) in the liver of rats exposed to cadmium (Cd). The rats received Cd in the diet at the concentration of 0, 1, and 5 mg Cd/kg and/or 0.1% aqueous AE (+) or not (-). Data are presented as mean ± SE for eight rats, except for seven animals in the AE, Cd_1_, and Cd_5_ group after 24 months. Statistically significant differences (ANOVA, Duncan’s multiple range test): * p < 0.05, ** *p* < 0.01, *** *p* < 0.001 vs. control group; ^†^ *p* < 0.05, ^††^ *p* < 0.01, ^†††^ *p* < 0.001 vs. respective group intoxicated with Cd alone; ^‡‡^ *p* < 0.01, ^‡‡‡^ *p* < 0.001 vs. group receiving AE alone; ^#^ *p* < 0.05, ^##^ *p* < 0.01, ^###^ *p* < 0.001 vs. respective group receiving the 1 mg Cd/kg diet (alone or with AE). Numerical values in bars disclose the percentage changes in comparison to the control group (↓, decrease; ↑, increase) or the respective group receiving Cd alone (↙, decrease; ↗, increase).

**Figure 6:**
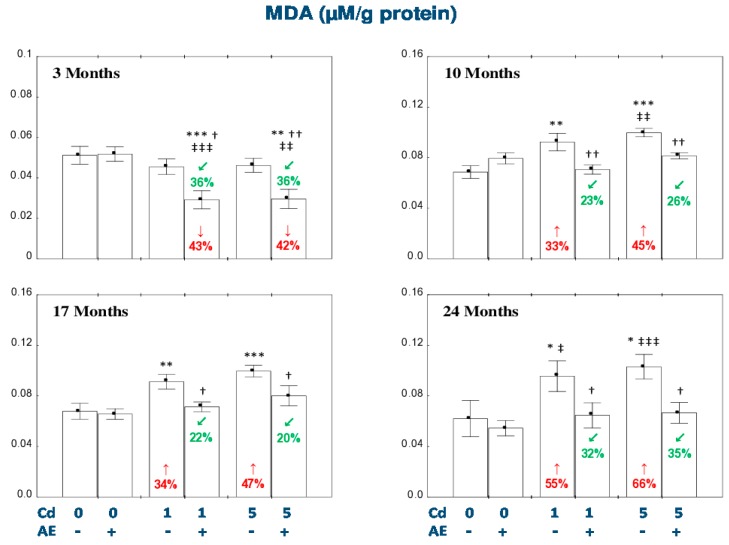
The effect of the extract from the berries of *Aronia melanocarpa* L. (AE) on the concentration of malondialdehyde (MDA) in the liver of rats exposed to cadmium (Cd). The rats received Cd in the diet at the concentration of 0, 1, and 5 mg Cd/kg and/or 0.1% aqueous AE (+) or not (-). Data are presented as mean ± SE for eight rats, except for seven animals in the AE, Cd_1_, and Cd_5_ group after 24 months. Statistically significant differences (ANOVA, Duncan’s multiple range test): * p < 0.05, ** *p* < 0.01, *** *p* < 0.001 vs. control group; ^†^ *p* < 0.05, ^††^ *p* < 0.01 vs. respective group intoxicated with Cd alone; ^‡^ *p* < 0.05, ^‡‡^ *p* < 0.01, ^‡‡‡^ *p* < 0.001 vs. group receiving AE alone. Numerical values in bars or above the bars disclose the percentage changes in comparison to the control group (↓, decrease; ↑, increase) or the respective group receiving Cd alone (↙, decrease).

The administration of AE alone for up to 24 months had no impact on TAS, TOS, OSI, and the concentration of MDA in the liver (Figure 5 and Figure 6). 

The exposure to the 1 mg Cd/kg diet resulted in a decrease in TAS and an increase in OSI after 10 and 17 months (by 28–92%; Figure 5). The liver TOS remained unchanged during the whole experiment (Figure 5). The administration of AE under the 10- and 17-month exposure to the 1 mg Cd/kg diet had no impact on the Cd-induced changes in TAS, but decreased TOS compared to the control group (by 38% and 29%, respectively) and Cd_1_ group (by 40% and 32%, respectively). Moreover, the extract intake completely protected against the increase in the value of OSI (Figure 5). The 24-month consumption of AE under the low-level exposure to Cd resulted in an increase in TAS and a decrease in OSI compared to the control group (by 18% and 23%, respectively) and Cd_1_ group (by 36% and 24%, respectively; Figure 5). In the animals treated with the 5 mg Cd/kg diet, TAS decreased (by 19%) after 10 months and TOS increased (by 51%) after 24 months of the investigation (Figure 5). The supplementation with AE during the moderate exposure to Cd totally prevented this xenobiotic influence on the liver TAS, whereas after 24 months this parameter was enhanced to the value higher compared to the control group and Cd_5_ group (by 43% and 54%, respectively; Figure 5). Apart of that, the consumption of the extract by the animals maintained on the 5 mg Cd/kg diet decreased the Cd alone-unchanged TOS after 10 and 17 months (by 19% and 20%, respectively) and partially prevented this xenobiotic-induced increase in TOS after 24 months of the experiment (Figure 5). In the rats intoxicated with the 5 mg Cd/kg diet, the value of OSI increased (by 17–61%) at each time point, whereas the co-administration of the extract completely protected against the development of oxidative stress evaluated based on the value of OSI (Figure 5). The low-level and moderate exposure to Cd for 10–24 months increased (by 33–66%) the concentration of MDA in the liver (Figure 6). The co-administration of the chokeberry extract provided entire protection against this effect of Cd. Moreover, the extract consumption at both levels of the 3-month exposure to Cd decreased the liver concentration of MDA compared to the control group (by 43% and 42%, respectively) and respective Cd group (by 36%; Figure 6). 

The ANOVA/MANOVA analysis showed that the effect of the consumption of AE on TAS, TOS, OSI, and MDA in the liver tissue under intoxication with the 1 and 5 mg Cd/kg diet, was the result of independent action of the extract ingredients (*F* = 4.519−101.2, *p* < 0.05−0.001) and/or their interaction with this heavy metal (*F* = 4.271–54.51, *p* < 0.05–0.001; Table S9). Nevertheless, the analysis revealed the lack of an independent effect of AE and its interaction with Cd (Table S9) on TAS in the Cd_5_ + AE group and TOS in the Cd_1_ + AE and Cd_5_ + AE groups after 10 months of the investigation, as well as MDA in the Cd_1_ + AE group after 10 and 24 months and in the Cd_5_ + AE group after 17 months of the study in spite of the evident impact of the extract administration under the exposure to this metal recognized on the basis of one-way analysis of variance (Figure 5 and Figure 6). 

The liver TAS after 10–24 months in the Cd_5_ + AE group was higher (by 21–36%) than in the Cd_1_ + AE group (Figure 5). Moreover, after 17 months TAS in the Cd_5_ group was higher (by 62%) compared to the Cd_1_ group (Figure 5). TOS in the Cd_5_ + AE group after 10 and 24 months was higher (by 37% and 41%, respectively) than in the Cd_1_ + AE group. Furthermore, the value of TOS in the Cd_5_ group after 24 months was higher compared to the Cd_1_ group (Figure 5). The extent of oxidative stress, evaluated based on the value of OSI, in the Cd_5_ group after 17 months was lower than in the Cd_1_ group (by 37%), while after 24 months oxidative stress in the Cd_5_ group and Cd_5_ + AE group was more severe (by 60% and 24%) than in the respective groups maintained on the diet containing 1 mg Cd/kg alone or with the extract (Figure 5). 

Positive correlations were noted between Cd concentration in the liver and TOS (*r* = 0.217, *p* < 0.01) and OSI (*r* = 0.145, *p* < 0.05), as well as the concentration of MDA (*r* = 0.323, *p* < 0.001) in this organ. 

### 3.6. Morphological Structure of the Liver 

The liver of the control group was *pinkish brown* in color, with a *soft consistency*, easily friable, and highly vascular. No differences in the macroscopic picture of the organ were noticed among all experimental groups.

The administration of AE alone had no impact on the absolute and relative weight of the liver (liver weight in grams and this organ weight expressed in calculation per 100 g of body weight, respectively; Table S10). Both parameters increased (by 17–30%) after the 10- and 24-month feedings with the diet containing 5 mg Cd/kg, but the co-administration of AE completely prevented these changes (Table S10).

**Figure 7:**
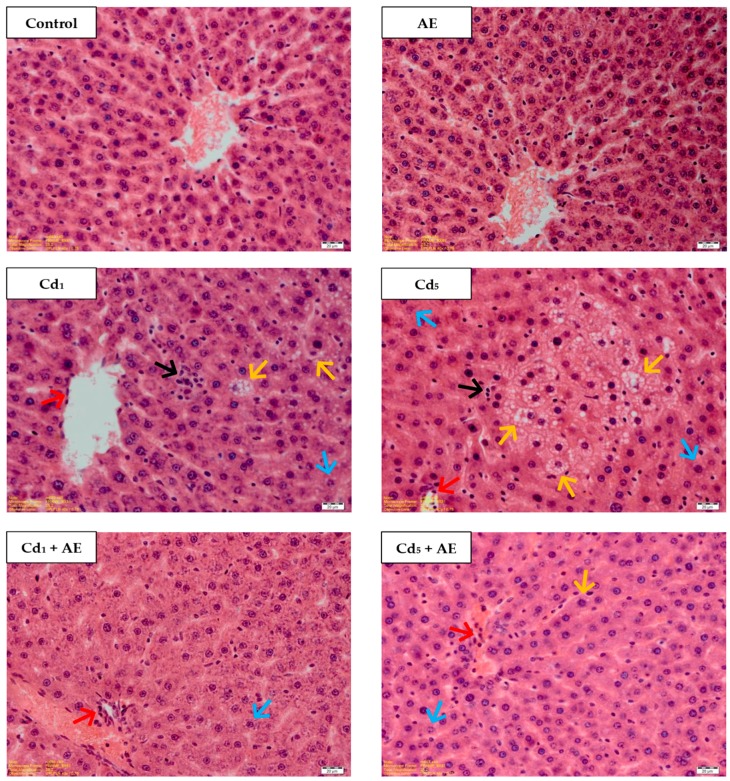
The effect of the extract from the berries of *Aronia melanocarpa* L. (AE) on the histological structure of the liver of rats exposed to cadmium (Cd) for 24 months. The rats received Cd in diet at the concentration of 0, 1, and 5 mg Cd/kg and/or 0.1% aqueous AE or not (H + E staining; ×400). Representative sections of the liver are presented. Control: normal liver in the control rats; AE: normal liver in the rats receiving 0.1% AE alone; Cd_1_ group: blurred trabecular structure—in some lobes, vacuolization and enlarged cells dimensions—in some lobes (↑), microvacuolar steatosis—in some lobes (↑), colliquative necrosis—in some lobes (**↑**), mononuclear cell infiltrations—in some lobes (↑); Cd_5_ group: blurred trabecular structure—in some lobes, vacuolization—in majority of cells, in some lobes (↑), colliquative necrosis—in some lobes (**↑**), microvacuolar steatosis—in some lobes (↑), mononuclear cell infiltrations—in some lobes, (↑); Cd_1_ + AE group: blurred trabecular structure, vacuolization and enlarged cells dimensions—in some cells in lobes (↑), mononuclear cell infiltrations (↑); Cd_5_ + AE group: blurred trabecular structure—in some lobes, microvacuolar steatosis—in some lobes (↑), vacuolization—in some lobes (↑), mononuclear cell infiltrations (↑).

The liver of the control animals, as well as these administered with AE alone showed a normal morphological picture evaluated under a light microscopy (Table 1, Figure 7). In the animals maintained on the diet containing 1 and 5 mg Cd/kg pathological changes in the morphological structure of the liver tissue such as blurred trabecular structure of the lobes, microvascular steatosis, colliguative necrosis, as well as vacuolization and enlarged dimensions of cells, and mononuclear cell infiltrations (indicating inflammatory changes) were observed (Table 1, Figure 7). The occurrence of the changes and their intensity depended on the level of exposure to Cd and its duration. At both levels of the treatment with this xenobiotic its unfavorable impact on the morphological structure of the liver was evident already after 3 months and it intensified with the duration of exposure (Table 1). 

In the rats receiving AE during the exposure to Cd the changes in the liver morphology of the same type, but of different intensity as in the animals treated with this metal alone, were observed (Table 1, Figure 7). The consumption of AE under the intoxication with this xenobiotic provided significant protection against the unfavorable impact of this xenobiotic on the liver microscopic structure. All of the pathological changes noted in the liver morphology were less intense in the animals co-administered with Cd and AE than in the respective groups of animals treated with Cd alone (Table 1, Figure 7). After 24 months of the treatment with the 1 and 5 mg Cd/kg diet (Cd_1_ and Cd_5_ groups), all the changes were observed in all rats or in at least 5 animals, whereas in the respective groups co-administered with AE and Cd (Cd_1_ + AE and Cd_5_ + AE groups), the changes were noted in at least five animals or were sporadic (occurred in one or two animals).

### 3.7. Effect of AE and/or Cd on the Activities of ALT and AST in the Serum

The administration of AE alone had no impact on the activities of ALT and AST in the serum, except for a decrease (by 33%) in the activity of ALT after 10 months of the experiment (Table 2). 

The intoxication with the 1 mg Cd/kg diet for 10, 17, and 24 months resulted in an increase in the activities of ALT (by 30–53%) and AST (by 27–75%), while the co-administration of AE provided complete protection against the impact of this xenobiotic except for ALT activity after 10 months and AST activity after 17 months (Table 2). In the rats receiving the diet containing 5 mg Cd/kg, the activity of ALT was increased (by 49–63%) from the 10-month of the study, whereas the activity of AST was elevated (by 32–41%) at each time point (Table 2). The administration of AE totally protected against these changes, except for the activity of AST after 10 months (Table 2). 

The effect of AE on the serum activities of ALT and AST of rats exposed to Cd was caused by independent action of the extract ingredients (*F* = 11.32−28.52, *p* < 0.01−0.001) and their interaction with this heavy metal (*F* = 4.275−14.46, *p* < 0.05–0.001; Table S11). However, the ANOVA/MANOVA analysis revealed the lack of an independent effect of AE and/or its interaction with Cd on the activity of AST in the Cd_1_ + AE group after 10 and 24 months, as well as in the Cd_5_ + AE group after 17 and 24 months (Table S11) in spite of the protective impact of the extract consumption under exposure to this metal revealed by one-way analysis of variance (Table 2). 

After 10 months of the study the activity of ALT in the Cd_5_ group was higher (by 26%) than in the Cd_1_ group, while its activity in the Cd_5_ + AE group was lower (by 36%) compared to the Cd_1_ + AE group (Table 2). The only difference in the activity of AST connected with the level of exposure to Cd was lower (by 19%) activity of this enzyme in the Cd_5_ group compared to the Cd_1_ group after 17 months of the investigation (Table 2).

Positive correlations occurred between Cd concentration in the liver and the serum activities of ALT (*r* = 0.148, *p* < 0.05) and AST (*r* = 0.171, *p* < 0.05).

## 4. Discussion

The present paper is the first study indicating the beneficial influence of a polyphenol-rich extract from *A. melanocarpa* L. berries on the oxidative/antioxidative balance in the liver and its ability to protect against the development of oxidative stress and this organ damage at low-level and moderate chronic intoxication with Cd corresponding to current environmental exposure to this xenobiotic [2,11,12,13,14,15,16,17,18,19]. A very important finding of this investigation is also revealing that Cd may destroy the oxidative/antioxidative balance and induce pathological changes in the morphological structure of the liver even at its low concentration in this organ (0.1447 ± 0.0093 µg/g), blood (0.1884 ± 0.0100 µg/L), and urine (0.2184 ± 0.0081 µg/g of creatinine) [28] comparable to the concentrations nowadays noted in the inhabitants of industrialized countries [11,12,13,14,15,16,17,18,19]. This finding indicates that the general population, due to common presence of Cd in the environment and food and its accumulation in the human body through the lifetime [6,7,8], may be at a real risk of development of oxidative stress in the liver and damage to this organ. At the same time it confirms the necessity of looking for effective ways to protect against oxidative stress and oxidative damage to various organs, including the liver.

The mechanisms of pro-oxidative action of Cd are widely reported [1] and thus they are not discussed in the present paper. Our findings show that Cd at both low-level and moderate exposure, may induce oxidative stress in the liver by weakening its enzymatic (SOD, CAT, GR, GPx, and GST) and nonenzymatic (TSH and GSH) antioxidative barrier and increasing the concentrations of pro-oxidants (GSSG, H_2_O_2_, MPO, and XOD; for more information on the role of oxidants and pro-oxidants see the Supplementary Material—Role of Antioxidants and Pro-oxidants). The decrease in the activities of antioxidative enzymes might result from interactions between Cd and metals such as Zn, Cu, manganese (Mn) (Zn, Cu-SOD, Mn-SOD), selenium (Se) (Se-GPx), and iron (Fe) (Fe-CAT) inhered in their active centers [1,39,40,41,42]. Such explanation seems very probable in the case of SOD as recent research by our team has revealed that maintenance of the rats on the diet containing 1 and 5 mg Cd/kg may disturb the liver homeostasis of Cu and Zn [27]. The fact that some indices of the enzymatic antioxidative barrier (SOD, CAT, GR, and GST) at some time points reached higher values in the animals fed the diet containing 5 mg Cd/kg than in the case of the 1 mg Cd/kg diet, and sometimes even higher than in the control group (CAT and GR) may be explained by starting the defense mechanisms [1,43]. It is vital to notice that antioxidants seem to be more susceptible to the unfavorable impact of Cd than oxidases since the activities of SOD and GPx and the concentration of GST were decreased already after 3 months of the treatment with the 1 mg Cd/kg diet, whereas the concentrations of MPO and XOD were elevated only after exposure longer than 3 months. The increase in the concentration of H_2_O_2_ in the liver might be the result of the decreased activities of enzymes which detoxify this compound such as CAT and GPx, as well as the increased concentration of XOD (Supplementary Material—Role of Antioxidants and Pro-oxidants). 

The Cd-caused destroying the oxidative/antioxidative balance resulted in a decrease in TAS and an increase in TOS of the liver and development of oxidative stress reflected in an elevated value of OSI and enhanced lipid peroxidation resulting in the pathological changes in the morphology of this organ. The accumulation of H_2_O_2_ and other ROS and FR in the liver cells may cause oxidative damage to the cellular and intracellular membranes (due to enhanced lipid peroxidation) leading to leakage of hepatic marker enzymes into the bloodstream [23,24,25,31,40,41,44,45], as it was reflected in our study in elevated activities of ALT and AST in the serum. The increase in the liver concentration of MDA as the effect of the low-level and moderate exposure to Cd together with positive correlations between Cd concentration and markers of oxidative stress indicates that the changes in the morphology of this organ and the serum activities of the liver marker enzymes were the result of this toxic metal-caused induction of oxidative stress. Because oxidative stress occurred earlier and was more potent in the animals maintained on the diet containing 5 mg Cd/kg compared to these feed with the 1 mg Cd/kg diet, the pathological changes in the liver were also more serious at the higher treatment. It should be emphasized that although at the low-level exposure to Cd oxidative stress was noted after 10 months, unfavorable changes of some parameters of the oxidative/antioxidative barrier were recorded already after 3 months of the intoxication. Taking into account the fact that morphological changes in hepatocytes, including oxidative damage to the cellular and intracellular membranes, result in disorders in the proper function of the liver [23,24,25,41,44,45,46]; based on the findings of the present study it can be concluded that low chronic exposure to Cd may also impair the function of this organ. The pathological changes in the morphological picture of the liver described by us are similar to these observed by other authors due to repeated intoxication with Cd [23,24,40,41,44,45]. However, the present study is the first report showing that such low exposure as 1 mg Cd/kg diet may lead to damage to the liver reflected in pathological changes in this organ morphological structure and enhanced activities of liver enzymes markers in the serum. 

The present research provided new and important data on Cd hepatotoxicity; however, our interest was focused first of all on the possible protective impact of prolonged consumption of AE on the oxidative/antioxidative status of the liver under chronic low-level and moderate treatment with Cd. Furthermore, the study allowed us to estimate the influence of the extract intake on the oxidative/antioxidative balance in this organ at very low exposure to this xenobiotic resulting from its trace, but unavoidable, presence in the standard diet (0.06 mg/kg in our experimental model [28,29]). Detailed analysis of the results of the present study in the animals receiving AE and fed with the diet without Cd addition has revealed that prolonged, even a lifetime, consumption of the extract in the daily dose of 31.1–154.7 mg/kg b.w. (corresponding to aronia polyphenols consumption ranging from 41.5 to 101.7 mg/kg b.w.) generally (except for temporary changes of some parameters) did not create a risk of destroying the oxidative/antioxidative balance of the liver. In the contrary, after 24 months of the extract consumption, the concentration of GSSG was lower, while the concentration of TSH and the ratio of GSH/GSSG were higher, this may indicate improved antioxidative properties of this organ. The risk of disturbing the balance between antioxidants and pro-oxidants is always a very important question related to the enhanced consumption of antioxidants, including polyphenol-rich products. The fact that the administration of AE under the low-level and moderate exposure to Cd offered important protection against pro-oxidative action of this xenobiotic, but had only slightly evident beneficial impact when it was administered alone may be explained by a lack of exposure to pro-oxidants via the standard laboratory diet and appropriate content of essential ingredients, including these possessing antioxidative properties. In the conditions of balance between pro-oxidants and antioxidants even prolonged supplementation with substances possessing antioxidative potential may have only minor, if any, impact on the oxidative/antioxidative status. This is in consistence with the results of other authors who showed that the administration of other antioxidants such as tetrahydrocurcumin [24], strawberry extract [25], and naringenin [44] alone does not affect oxidative/antioxidative balance of the liver in the organism not exposed to pro-oxidative agents. However, the present study has revealed, first of all, that the intake of AE is capable of improving the oxidative/antioxidative status and completely prevent the development of oxidative stress in this organ under both low-level and moderate lifetime exposure to Cd. The beneficial impact of the consumption of AE was connected not only with an enhanced antioxidative potential of the liver, but also with decreased concentrations of oxidases and H_2_O_2_ in this organ. It is important to underline that although the impact of Cd on the values of OSI (reflecting the intensity of oxidative stress) differed, depending on the duration and the level of exposure, the extract co-administration was capable of completely protecting the liver from the development of oxidative stress irrespective of the intensity of intoxication with this xenobiotic. What is more, the beneficial effect of AE was reflected in a decrease in the liver concentration of MDA and the serum activities of ALT and AST, as well as in its ability to protect from these metal-induced changes in this organ morphology.

The influence of AE on the liver under the treatment with Cd may be explained by a direct effect of the extract resulting from its strong antioxidative potential [4,5,26] and an indirect action related to interactions between ingredients of the chokeberry extract and this toxic element [28,32,33,34]. Both effects might be due to first of all the high abundance of polyphenolic compounds [4,5,27,28,33,34] which may serve as hydrogen donors able to inactivate FR and ROS, terminate radical chain reactions such as lipid peroxidation, and play an important function in the regeneration of nonenzymatic antioxidants [3,42,47]. It is vital to emphasize that the beneficial influence of AE may also be due to the presence in the extract of ingredients other than polyphenols with proven ability to combat Cd toxicity such as vitamins C and E, β-carotene, triterpens, fiber, pectin, and essential microelements such as Fe, Zn, and Se [3,4,40,41,47,48,49,50]. Taking into account the recent findings of our research team on the impact of AE on the body status of Zn and Cu at the conditions of low-level and moderate intoxication with Cd, and these bioelements binding to metallothionein (MT) [27], the beneficial effect of the extract might also be mediated by this low molecular weight (6–7 kDa), rich in thiol groups (-SH groups), metal binding protein, and the ability to maintain the pool of MT-unbound Zn and Cu in this organ at the proper level. Such explanation seems to be possible as the extract administration for 17 months under the both low-level and moderate exposure to Cd completely prevented this heavy metal induced inhibition of the activity of SOD and even enhanced this enzyme activity in the case of the higher 24-month treatment.

Our previous reports on the impact of AE on the body burden of Cd [28], together with the positive correlations, noted in the present study, between Cd concentration in the liver and the values of TOS and OSI, as well as the concentrations of H_2_O_2_ and MDA, allow for the conclusion that the favorable effects of AE may also be explained by the ability of chokeberry ingredients to reduce the body and liver burden of Cd by decreasing this xenobiotic absorption from the gastrointestinal tract and increasing its urinary excretion. The presence of -OH groups and carbonyl groups in the structure of polyphenols, especially the occurrence of 3’4’-dihydroxyl groups in the B ring, simultaneous occurrence of 5-hydroxyl group in the A ring, and 4-carbonyl group in the C ring as well as the 3-hydroxyl and 4-carbonyl groups in the C ring, enables polyphenols to bind ions of metals, including Cd^2+^ [33,34,51].

The present paper is the only data on the influence of chokeberry extract on the oxidative/antioxidative balance, morphology, and functional status of the liver under the conditions of low-level and moderate exposure to Cd. However, some experimental investigations revealed the preventive effect of rich in polyphenolic compounds extracts from green tea, strawberry, blueberry, ginger, and olive oil [23,25,52,53,54,55], as well as single polyphenols such as curcumin and naringenin [24,44] in protection from this metal-induced oxidative stress in the liver and hepatocytes damage. Since oxidative stress has been recognized as the crucial pathway of this xenobiotic-induced injury to the liver [1,23,24,25,40,44,45,52,55], the improvement in the antioxidative potential of hepatocytes seems to be the key mechanism in combating the negative effects of the action of this heavy metal. The fact that AE prevents the development of Cd-induced oxidative stress and lesions in liver morphology and function allows us to suspect that the consumption of chokeberry extract may protect this organ from oxidative damage.

The present study is a part of comprehensive researches evaluating the possibility of using *A. melanocarpa* L. berries in protection of the organism from detrimental effects of the exposure to Cd. The results of this investigation together with the previously reported findings suggest that consumption of chokeberry products may provide protection for the target organs of this metal toxicity. These reports have not only scientific value but also an important practical application, since the consumption of aronia berries is widely recommended due to their multidirectional health-promoting properties [4,5]. Moreover, the beneficial influence of AE on the liver under exposure to Cd allows us to suspect that the consumption of the extract may also protect this organ from the damaging influence of other xenobiotics characterized by pro-oxidative properties.

We are aware not only of the accomplishments, but also of the limitations of our investigation. Since women are more susceptible to Cd toxicity than man, the experimental model used in this study consists with female rats, therefore our results refer to the female liver. Thus, further research involving an evaluation of AE on the male liver is needed. Moreover, it is necessary to examine to what extent chokeberry extract can protect against oxidative damage to the liver and explain the involvement of antioxidative properties of the extract in the mechanisms of its protective action on this organ at the conditions of low-level and moderate chronic exposure to Cd. We have undertaken research in this direction and their results will be published soon. 

## 5. Conclusions

In conclusion, this study demonstrated, for the first time, that the extract from the berries of *A. melanoccarpa* L. offers protection from the development of oxidative stress in the liver and improves this organ morphology in rats at low-level and moderate treatment with Cd reflecting the environmental exposure of humans. The protective action of AE may be explained by high occurrence of numerous beneficial compounds, mainly polyphenols, able to scavenge FR and ROS, and suppress the activity of oxidases, as well as by the presence in the extract of ingredients possessing the ability to chelate Cd^2+^. Although further studies have to be undertaken in this subject, based on the results of this investigation, it can be concluded that aronia berries and their products may be very promising natural agents to be applied in the protection from oxidative damage of the liver in humans exposed to this toxic metal. This is the most important and practically useful, but not the only achievement of our research. Very significant and also of practical usefulness finding of the study is revealing the risk of the development of oxidative stress and the liver injury even at low-level repeated exposure to Cd, as well as providing evidence that prolonged enhanced consumption of AE under exposure to only trace amounts of this metal do not create a risk of destroying the balance between antioxidants and pro-oxidants.

## Figures and Tables

**Table 1:** The effect of the extract from the berries of *Aronia melanocarpa* L. (AE) on the liver morphology of rats exposed to cadmium (Cd) ^1,2^.

Group	Blurred Trabecular Structure of the Lobes	Microvascular Steatosis	Colliquative Necrosis	Vacuolization, Enlarged Dimensions of Cells	Mononuclear Cell Infiltrations
**3 Months**					
Control	–	–	–	–	–
AE	–	–	–	–	–
Cd_1_	± ^b^	–	–	± ^d^	± ^d^
Cd_1_ + AE	± ^b^	–	–	± ^b^	± ^d^
Cd_5_	± ^d^	± ^d^	± ^b^	± ^d^	± ^b^
Cd_5_ + AE	± ^b^	± ^b^	–	± ^b^	± ^b^
**10 Months**					
Control	–	–	–	–	–
AE	–	–	–	–	–
Cd_1_	± ^d^	± ^b^	± ^a^	± ^b^	± ^b^
Cd_1_ + AE	± ^b^	–	–	± ^a^	± ^b^
Cd_5_	+ ^d^	± ^d^	± ^d^	± ^d^	± ^d^
Cd_5_ + AE	± ^d^	± ^b^	± ^b^	± ^b^	± ^b^
**17 Months**					
Control	–	–	–	–	–
AE	–	–	–	–	–
Cd_1_	+ ^d^	+ ^d^	+ ^d^	+ ^d^	+ ^d^
Cd_1_ + AE	± ^d^	± ^d^	± ^d^	+ ^b^	± ^d^
Cd_5_	++ ^d^	+ ^d^	++ ^d^	++ ^d^	+ ^d^
Cd_5_ + AE	+ ^d^	± ^d^	+ ^d^	+ ^c^	± ^d^
**24 Months**					
Control	–	–	–	–	–
AE	–	–	–	–	–
Cd_1_	+ ^d^	++ ^d^	+ ^d^	++ ^d^	+ ^d^
Cd_1_ + AE	± ^d^	+ ^d^	± ^d^	+ ^b^	± ^d^
Cd_5_	++ ^d^	++ ^d^	++ ^d^	++ ^b^	+ ^d^
Cd_5_ + AE	+ ^d^	+ ^d^	+ ^d^	+ ^b^	± ^d^

^1^ The rats received the 0.1% aqueous AE and Cd in diet at the concentration of 1 or 5 mg/kg for 3–24 months. ^2^ The following criteria were used to scoring the presence or absence of changes in the liver morphology: ++, a change was found in some lobes, in all animals of a group; +, a change was found in some lobes in a majority of animals of a group (occurred in at least five animals); ±, a change was sporadic in a group (occurred in one or two animals); –, a change was absent in all animals of a group; ^a^ a change was present in single cells in a group; ^b^ a change was found in some cells in lobes; ^c^ a change occurred in the majority of cells in lobes; ^d^ a change was found in almost all cells in lobes.

**Table 2:** The effect of the extract from the berries of *Aronia melanocarpa* L. (AE) on the activities of alanine aminotransferase (ALT) and *aspartate transaminase* (AST) in the serum of rats exposed to cadmium (Cd) ^1,2^.

Group	Duration of the Experiment			
	3 Months	10 Months	17 Months	24 Months
**ALT (U/L)**				
Control	24.19 ± 1.306	22.55 ± 1.308	15.95 ± 1.686	24.11 ± 3.003
AE	22.70 ± 1.993	15.05 ± 1.004 *	16.22 ± 1.453	31.86 ± 5.217
Cd_1_	21.74 ± 3.480	29.29 ± 2.260 * ^‡‡‡^	24.44 ± 2.960 ** ^‡‡^	33.65 ± 3.825 *
Cd_1_ + AE	21.82 ± 2.020	30.25 ± 3.320 * ^‡‡‡^	10.54 ± 0.397 ^††† ‡^	22.61 ± 2.184 ^† ‡^
Cd_5_	23.15 ± 3.442	36.77 ± 4.278 *** ^‡‡‡ #^	24.64 ± 2.226 ** ^‡‡^	35.92 ± 2.078 *
Cd_5_ + AE	23.35 ± 3.506	19.50 ± 0.846 ^††† ##^	12.79 ± 1.314 ^†††^	20.44 ± 1.006 ^†† ‡^
**AST (U/L)**				
Control	82.28 ± 5.983	62.98 ± 2.645	79.48 ± 8.155	45.32 ± 4.260
AE	96.14 ± 4.549	64.39 ± 4.516	94.32 ± 9.591	47.54 ± 2.612
Cd_1_	96.24 ± 7.035	80.68 ± 7.370 *	139.02 ± 10.35 *** ^‡‡‡^	57.33 ± 4.174 *
Cd_1_ + AE	101.5 ± 6.851	77.70 ± 7.296	127.5 ± 7.253 *** ^‡^	51.65 ± 5.743
Cd_5_	113.6 ± 5.391 **	84.10 ± 7.487 *	112.37 ± 8.591 * ^#^	60.02 ± 2.004 *
Cd_5_ + AE	93.92 ± 7.822	83.37 ± 7.533 *	104.10 ± 7.918	51.58 ± 2.396

^1^ The rats received the 0.1% aqueous AE and Cd in diet at the concentration of 1 or 5 mg/kg for 3–24 months. ^2^ Data are presented as mean ± SE for eight rats, except for seven animals in the AE, Cd_1_, and Cd_5_ group after 24 months. Statistically significant differences (ANOVA, Duncan’s multiple range test): * p < 0.05, ** *p* < 0.01, *** *p* < 0.001 vs. control group; ^†^ *p* < 0.05, ^††^ *p* < 0.01, ^†††^ *p* < 0.001 vs. respective group intoxicated with Cd alone; ^‡^ *p* < 0.05, ^‡‡^ *p* < 0.01, ^‡‡‡^ *p* < 0.001 vs. group receiving AE alone; ^#^ *p* < 0.05, ^##^ *p* < 0.01 vs. respective group receiving the 1 mg Cd/kg diet (alone or with AE).

## Supplementary Materials

The following are available online at http://www.mdpi.com/2072-6643/11/1/21/s1. Supplementary Material—Analytical Quality of Measurements, Supplementary Material—Role of Antioxidants and Pro-oxidants, Table S1: The concentration of cadmium (Cd) in the blood, liver, and urine of rats receiving the extract from the berries of *Aronia melanocarpa* L. (AE) and/or Cd, Table S2: Polyphenolic composition of the extract from the berries of *Aronia melanocarpa* L. (AE), Table S3: The intake of cadmium (Cd) and the extract from the berries of *Aronia melanocarpa* L. (AE) in particular experimental groups, Table S4: Main and interactive effects of cadmium (Cd) and the extract from the berries of *Aronia melanocarpa* L. (AE) on the activities of superoxide dismutase (SOD) and catalase (CAT) in the liver of rats, Table S5: Main and interactive effects of cadmium (Cd) and the extract from the berries of *Aronia melanocarpa* L. (AE) on the activities of glutathione peroxidase (GPx) and glutathione reductase (GR) and the concentration of glutathione S-transferase (GST) in the liver of rats, Table S6: Main and interactive effects of cadmium (Cd) and the extract from the berries of *Aronia melanocarpa* L. (AE) on the concentrations of reduced glutathione (GSH), oxidized glutathione (GSSG), and the ratio of GSH/GSSGS, as well as the concentration of total thiol groups (TSH) in the liver of rats, Table S7: Effect of the extract from the berries of *Aronia melanocarpa* L. (AE) on the concentration of thioredoxin (Trx) in the liver of rats exposed to cadmium (Cd), Table S8: Main and interactive effects of cadmium (Cd) and the extract from the berries of *Aronia melanocarpa* L. (AE) on the concentrations of hydrogen peroxide (H_2_O_2_), myeloperoxidase (MPO), and xanthine oxidase (XOD) in the liver of rats, Table S9: Main and interactive effects of cadmium (Cd) and the extract from the berries of *Aronia melanocarpa* L. (AE) on total antioxidative status (TAS), total oxidative status (TOS) and the index of oxidative stress (OSI) in the liver of rats, Table S10: The effect of the extract from the berries of *Aronia melanocarpa* L. (AE) on the absolute and relative weight of the liver of rats exposed to cadmium (Cd), Table S11: Main and interactive effects of cadmium (Cd) and the extract from the berries of *Aronia melanocarpa* L. (AE) on the activities of alanine aminotransferase (ALT) and aspartate transaminase (AST) in the serum of rats, Figure S1: The effect of the extract from the berries of *Aronia melanocarpa* L. (AE) on the concentration and content of cadmium (Cd) in the liver of rats exposed to this metal.

## Abbreviations

AE	extract from the berries of *Aronia melanocarpa* L.
ALT	alanine aminotransferase
ANOVA	a one-way analysis of variance
ANOVA/MANOVA	a two-way analysis of variance
AST	*aspartate transaminase*
CAT	catalase
Cd	cadmium
Cd^2+^	divalent ion of cadmium
Cu	copper
DTNB	5,5’-dithio-bis-2-nitrobenzoic acid
ELISA	enzyme-linked immunosorbent assay
FR	free radicals
GPx	glutathione peroxidase
GR	glutathione reductase
GSH	reduced glutathione
GSSG	oxidized glutathione
GST	glutathione S-transferase
H + E	hematoxylin and eosin
H_2_O_2_	hydrogen peroxide
MDA	malondialdehyde
MPO	myeloperoxidase
MT	metallothionein
NADPH	reduced nicotinamide adenine dinucleotide phosphate
NADP^+^	oxidized nicotinamide adenine dinucleotide phosphate
-OH group	hydroxyl group
OSI	oxidative stress index
O_2_^**·**-^	superoxide radicals
r	correlation coefficient
ROS	reactive oxygen species
SD	standard deviation
SE	standard error
SOD	superoxide dismutase
-SH group	thiol group
TAS	total antioxidative status
TNT	5-thio-2-nitrobenzoic acid
Trx	thioredoxin
TOS	total oxidative status
TSH	total thiol groups
XOD	xanthine oxidase
Zn	zinc
