# Supplementary material for: Extract from Aronia melanocarpa L. Berries Prevents Cadmium-Induced Oxidative Stress in the Liver: A Study in A Rat Model of Low-Level and Moderate Lifetime Human Exposure to this Toxic Metal
Source: Nutrients. 2018 Dec 21;11(1):21. doi: 10.3390/nu11010021 (PMC6357096; doi:10.3390/nu11010021)
Supplement: Supplementary file 1 [file nutrients-11-00021-s001.pdf]

Supplementary Materials

Extract from *Aronia melanocarpa* L. Berries Prevents Cadmium-induced Oxidative Stress in the Liver: a Study in a Rat Model of Low-level and Moderate Lifetime Human Exposure to this Toxic Metal

Magdalena Mężyńska [1,*], Małgorzata M. Brzóska [1,*], Joanna Rogalska [1], Barbara Piłat-Marcinkiewicz [2]

[1] Department of Toxicology, Medical University of Bialystok, Adama Mickiewicza 2C street, 15-222 Bialystok, Poland; joanna.rogalska@umb.edu.pl

[2] Department of Histology and Embryology, Medical University of Bialystok, Jerzego Waszyngtona 13 street, 15-269 Bialystok, Poland; barbara.pilat-marcinkiewicz@umb.edu.pl

* Correspondence: mmezynska1@student.umb.edu.pl; malgorzata.brzoska@umb.edu.pl; tel.: +48 85 7485604; fax: +48 85 7485834

Supplementary Material – Analytical Quality of Measurements

The precision of the measurements, expressed as intra-assay coefficient of variation (CV), for superoxide dismutase (SOD), catalase (CAT), glutathione peroxidase (GPx), and glutathione reductase (GR) was <3.4%, 2.7%, 4.9%, and 6%, respectively, whereas the inter-assay CV for SOD was <3%.

The intra-assay CV for reduced glutathione (GSH) and oxidized glutathione (GSSG) was <4% and for total thiol groups (TSH) was <3.4%, while the inter-assay CV was <1.5% for GSH and <3% for GSSG.

The liver concentrations of glutathione S-transferase (GST), thioredoxin (TRx), xanthine oxidase (XOD), and myeloperoxidase (MPO) were determined with the intra-assay CV <5%, 2%, 2.3%, and 4%, respectively. The inter-assay CV was <5% for GST, <5% for Trx, <3.3% for XOD, and <4.4% for MPO.

The analytical quality of the assay of total antioxidative status (TAS) and total oxidative status (TOS) was determined by the measurement of these parameters in control samples included in the kits and based on the intra- and inter-assay CV. The certified values of TAS in the control samples were 172–232 and 239–323 µmol/L, while the values quantified by us reached 205.5 ± 12.5 and 271.5 ± 4.5 µmol/L (mean ± SD), respectively. The values of TOS in the control samples were 169–282 and 407–678 µmol/L, while the values determined by us amounted to 218.3 ± 20 and 609.6 ± 27.2 µmol/L (mean ± SD), respectively. The intra-assay CV was <4.6% for TAS and 4.2% for TOS, whereas the inter-assay CV was <4% and <4.5%, respectively.

Supplementary Material – Role of Antioxidants and Pro-oxidants

SOD, CAT, GPx, GR, and GST are the main enzymatic free-radical scavengers in cells [1,2]. SOD is the enzyme which catalyses the dismutation of superoxide radical ($O_2^{\cdot -}$), whereas CAT and GPx are engaged in the detoxification of H_2O_2 [2]. GPx, GR, and GST are enzymes involved in the metabolism of GSH. GPx promotes the oxidation of GSH to GSSG, while GR is accountable for the reduction of GSSG [2]. As the main non-enzymatic antioxidant GSH plays an important function in the inactivation of organic and inorganic free radicals (FR) and xenobiotics, including heavy metals such as cadmium (Cd) [2]. Due to the presence of -SH group, GSH may bind ions of this metal (Cd^{2+}) into an inert complex excreted in the bile [2]. Trx being a protein with two -SH groups plays similar role as GSH [2].

MPO is an enzyme occurring in the membranes of neutrophils which catalyses the synthesis of podchloric acid with strong oxidative and antibacterial abilities [3], whereas XOD takes part in the metabolism of purines [4]. Reactions catalysed by both MPO and XOD are the source of reactive oxidants, including H_2O_2 and $O_2^{\cdot -}$ [3,4]. Enhanced concentration of H_2O_2 in cells has very negative consequences,

because this compound in the presence of ions of transitive metals such as iron and copper may generate a highly reactive hydroxyl radical ($HO^{\cdot}$) via the Fenton reaction [2]. The accumulation of H_2O_2 and other reactive oxygen species and FR may have detrimental effects on cellular metabolism, respiration, energy production, signalling pathways, and may cause damage to the cellular and intracellular membranes and key macromolecules (proteins, lipids, and DNA) [1,2,5–9]. Moreover, the Cd-induced decline in the concentration of GST, an enzyme of phase II detoxification which couples toxic metabolites with GSH, may change the metabolism of various substances, and increase their toxicity [2,10].

Table S1. The concentration of cadmium (Cd) in the blood, liver, and urine of rats receiving the extract from the berries of *Aronia melanocarpa* L. (AE) and/or Cd [1,2].

Group	Duration of the Experiment			
	**3 Months**	**10 Months**	**17 Months**	**24 Months**
	**Blood (μg/L)**			
Control	0.0691 ± 0.0079	0.0860 ± 0.0091	0.0743 ± 0.0052	0.0834 ± 0.0043
AE	0.0752 ± 0.0046	0.0802 ± 0.0087	0.0718 ± 0.0099	0.0861 ± 0.0038
Cd_1	0.1884 ± 0.0100*	0.1792 ± 0.0198*	0.2425 ± 0.0167***	0.2330 ± 0.0143**
Cd_1 + AE	0.1887 ± 0.0124*	0.1844 ± 0.0137*	0.2375 ± 0.0166***	0.2188 ± 0.0143**
Cd_5	1.0236 ± 0.066***	0.9394 ± 0.0439***	1.0339 ± 0.0266***	1.0467 ± 0.0508***
Cd_5 + AE	0.8298 ± 0.0544*** †††	0.7948 ± 0.0454*** †††	0.9319 ± 0.0413*** †††	0.8503 ± 0.0600*** †††
	**Liver (μg/g)**			
Control	0.0348 ± 0.0026	0.0231 ± 0.0011	0.0138 ± 0.0016	0.0137 ± 0.0015
AE	0.0312 ± 0.0014	0.0202 ± 0.0014	0.0145 ± 0.0019	0.014 ± 0.0016
Cd_1	0.1447 ± 0.0093***	0.199 ± 0.028***	0.211 ± 0.019***	0.364 ± 0.025***
Cd_1 + AE	0.0968 ± 0.007*** †††	0.179 ± 0.0179***	0.1913 ± 0.0168***	0.2275 ± 0.0335*** †
Cd_5	0.912 ± 0.053***	1.617 ± 0.112***	2.449 ± 0.178***	2.755 ± 0.089***
Cd_5 + AE	0.7427 ± 0.0045*** †††	1.4424 ± 0.0916*** †††	1.8479 ± 0.3144*** ††	2.4911 ± 0.1342*** ††
	**Urine (μg/g of creatinine)**			
Control	0.1387 ± 0.0103	0.1304 ± 0.0080	0.1491 ± 0.0103	0.1337 ± 0.0154
AE	0.1321 ± 0.0046	0.1364 ± 0.0043	0.1445 ± 0.0116	0.1357 ± 0.0070
Cd_1	0.2184 ± 0.0081**	0.1809 ± 0.0194**	0.2096 ± 0.0215*	0.2053 ± 0.0155*
Cd_1 + AE	0.2193 ± 0.0171**	0.1913 ± 0.0113**	0.2143 ± 0.0268*	0.2084 ± 0.0105*
Cd_5	0.5008 ± 0.0234***	0.4002 ± 0.0480***	0.4147 ± 0.0390***	0.4104 ± 0.0198***
Cd_5 + AE	0.6064 ± 0.0374*** †††	0.4997 ± 0.0400*** †††	0.5773 ± 0.0420*** †††	0.4994 ± 0.0438*** †††

[1] The rats received the 0.1% aqueous AE and Cd in diet at the concentration of 1 or 5 mg/kg for 3–24 months.

[2] Data are represented as mean ± SE for 8 rats (except for 7 animals in the AE, Cd_1, and Cd_5 groups after 24 months). Statistically significant differences (ANOVA, Duncan's multiple range test) compared to the control group (* $p < 0.05$, ** $p < 0.01$, *** $p < 0.001$) and respective group receiving Cd alone († $p < 0.05$, †† $p < 0.01$, ††† $p < 0.001$) are marked.

Table S2. Polyphenolic composition of the extract from the berries of *Aronia melanocarpa* L. (AE) [1,2].

Compound	Concentration (mg/g)
Total polyphenols	612.40 ± 3.33
Total anthocyanins	202.28 ± 1.28
Total proanthocyanidins	129.87 ± 1.12
Total phenolic acids	110.92 ± 0.89
Total flavonoids	21.94 ± 0.98
Chlorogenic acid	68.32 ± 0.08
Cyanidin 3-O-β-galactoside	80.07 ± 1.05
Cyanidin 3-O-α-arabinoside	33.21 ± 0.01
Cyanidin 3-O-β-glucoside	3.68 ± 0.01

[1] The concentrations of total polyphenols, total phenolic acids, flavonoids, proantocyanidins, and anthocyanins in the aronia extract by Adamed Consumer Healthcare were determined spectrophotometrically [11,12]. Ultra Performance Liquid Chromatography was used to evaluate the polyphenolic profile of the extract and quantificate chlorogenic acid and anthocyanins (cyanidin 3-O-β-galactoside, cyanidin 3-O-α-arabinoside, and cyanidin 3-O-β-glucoside [11].

[2] Data are represented as mean ± SE for 3 independent measurements.

Table S3. The intake of cadmium (Cd) and the extract from the berries of *Aronia melanocarpa* L. (AE) in particular experimental groups [1,2,3,4].

Group	Duration of the Experiment			
	**3 Months**	**10 Months**	**17 Months**	**24 Months**
	**Daily Cd Intake (μg/kg b.w.)**			
Control	4.709 ± 0.067	2.716 ± 0.060	2.582 ± 0.047	2.597 ± 0.051
AE	4.615 ± 0.065	2.935 ± 0.063	2.410 ± 0.046	2.764 ± 0.059
Cd_1	77.50 ± 1.264***	43.85 ± 0.60***	43.41 ± 0.64***	43.22 ± 0.85***
Cd_1 + AE	80.93 ± 1.06***	46.90 ± 0.84***	40.15 ± 0.77***	48.12 ± 0.94***
Cd_5	383.65 ± 4.44***	235.78 ± 2.55***	214.54 ± 2.83***	253.75 ± 8.63***
Cd_5 + AE	385.92 ± 4.91***	239.66 ± 1.43***	208.75 ± 3.04***	252.06 ± 9.29***
	**Daily AE Intake (mg/kg b.w.)**			
AE	139.22 ± 1.71	94.733 ± 2.84	80.975 ± 1.72	72.942 ± 2.37
Cd_1 + AE	148.26 ± 2.20	92.666 ± 1.63	83.662 ± 1.16	74.928 ± 2.37
Cd_5 + AE	140.58 ± 1.96	99.077 ± 2.70	85.237 ± 1.74	73.414 ± 3.15
	**Daily Polyphenols Intake (mg/kg b.w.)**			
AE	91.53 ± 1.12	62.29 ± 1.87	53.22 ± 1.14	47.96 ± 1.56
Cd_1 + AE	97.47 ± 1.45	60.92 ± 1.07	55.00 ± 0.76	49.26 ± 1.57
Cd_5 + AE	92.41 ± 1.30	65.14 ± 1.77	56.02 ± 1.15	48.26 ± 2.07

[1] The rats received the 0.1% aqueous AE and Cd in diet at the concentration of 1 or 5 mg/kg for 3–24 months.

[2] Data are represented as mean ± SE for 8 rats (except for 7 animals in the AE, Cd_1, and Cd_5 groups after 24 months).

[3] The intake of Cd in the control group and the group receiving AE alone was calculated based on this metal concentration determined by us in the standard diet (0.0584 mg/kg) [11], whereas this xenobiotic intake in the groups exposed to Cd (1 or 5 mg Cd/kg) was calculated based on its concentration in the feed declared by the manufacturer. Statistically significant differences (ANOVA, Duncan's multiple range test) compared to the control group (*** $p < 0.001$) are marked.

[4] The intake of polyphenols was calculated assuming that the AE contained 65.74% of these compounds (manufacturer's data). The daily consumption of the extract and polyphenolic compounds by rats during the experiment ranged from 63.1 to 159.1 mg/kg b.w. and from 41.5 to 104.6 mg/kg b.w., respectively [11]. The intake of AE and polyphenols in the control group, Cd_1 group, and Cd_5 group was 0. There were no statistically significant differences ($p > 0.05$; ANOVA, Duncan's multiple range test) in the intake of the extract and polyphenolic compounds between the AE, Cd_1 + AE, and Cd_5 + AE groups.

Table S4. Main and interactive effects of cadmium (Cd) and the extract from the berries of *Aronia melanocarpa* L. (AE) on the activities of superoxide dismutase (SOD) and catalase (CAT) in the liver of rats [1,2].

Duration (months)	Parameter	Exposure to 1 mg Cd/kg diet			Exposure to 5 mg Cd/kg diet		
		Main effect of Cd	Main effect of AE	Main effect of Cd + AE	Main effect of Cd	Main effect of AE	Main effect of Cd + AE
**3**	**SOD**	23.85***	NS	NS	-	-	-
	**CAT**	-	-	-	18.95***	NS	NS
**10**	**SOD**	32.75***	NS	NS	-	-	-
	**CAT**	NS	6.852*	NS	-	-	-
**17**	**SOD**	11.21**	NS	NS	NS	4.370*	7.152*
	**CAT**	5.191*	5.337*	NS	42.79***	NS	5.415*
**24**	**SOD**	-	-	-	NS	4.650*	8.478**
	**CAT**	29.48***	NS	7.723*	NS	NS	4.961*

[1] The rats received the 0.1% aqueous AE and Cd in diet at the concentration of 1 or 5 mg/kg for 3–24 months.

[2] The results of the ANOVA/MANOVA analysis are presented as *F* values and the level of statistical significance (p). *F* values having $p < 0.05$ were considered statistically significant (* $p < 0.05$, ** $p < 0.01$, *** $p < 0.001$). NS – not statistically significant ($p > 0.05$). In the case when a one way-analysis of variance (ANOVA, Duncan's multiple range test) revealed any influence of the co-administration of Cd and AE on the investigated parameter, a two-way analysis of variance (ANOVA/MANOVA, test *F*) was conducted in aim to discern possible interactive and independent impact of Cd and AE on this parameter.

Table S5. Main and interactive effects of cadmium (Cd) and the extract from the berries of *Aronia melanocarpa* L. (AE) on the activities of glutathione peroxidase (GPx) and glutathione reductase (GR) and the concentration of glutathione S-transferase (GST) in the liver of rats [1,2].

Duration (months)	Parameter	Exposure to 1 mg Cd/kg diet			Exposure to 5 mg Cd/kg diet		
		Main effect of Cd	Main effect of AE	Main effect of Cd + AE	Main effect of Cd	Main effect of AE	Main effect of Cd + AE
**3**	**GPx**	9.127*	8.329*	NS	61.56***	NS	NS
	**GR**	NS	21.72***	NS	57.36***	NS	13.24**
	**GST**	5.970*	NS	NS	16.3***	NS	NS
**10**	**GPx**	NS	NS	6.242*	9.702**	NS	11.77**
	**GR**	5.076*	NS	NS	8.671**	NS	NS
	**GST**	15.17***	9.913**	8.917**	22.58***	NS	NS
**17**	**GPx**	23.88***	NS	7.996*	12.96**	NS	8.988**
	**GR**	-	-	-	16.07***	NS	NS
	**GST**	NS	NS	16.48***	NS	NS	12.11**
**24**	**GPx**	51.06***	NS	12.78**	18.46***	NS	30.31***
	**GR**	NS	34.18***	16.08***	5.790*	10.81**	NS
	**GST**	-	-	-	NS	6.998*	10.43**

[1] The rats received the 0.1% aqueous AE and Cd in diet at the concentration of 1 or 5 mg/kg for 3–24 months.

[2] The results of the ANOVA/MANOVA analysis are presented as F values and the level of statistical significance (p). F values having $p < 0.05$ were considered statistically significant (* $p < 0.05$, ** $p < 0.01$, *** $p < 0.001$). NS – not statistically significant ($p > 0.05$). In the case when a one way-analysis of variance (ANOVA, Duncan's multiple range test) revealed any influence of the co-administration of Cd and AE on the investigated parameter, a two-way analysis of variance (ANOVA/MANOVA, test F) was conducted in aim to discern possible interactive and independent impact of Cd and AE on this parameter.

Table S6. Main and interactive effects of cadmium (Cd) and the extract from the berries of *Aronia melanocarpa* L. (AE) on the concentrations of reduced glutathione (GSH), oxidized glutathione (GSSG), and the ratio of GSH/GSSGS, as well as the concentration of total thiol groups (TSH) in the liver of rats [1,2].

Duration (months)	Parameter	Exposure to 1 mg Cd/kg diet			Exposure to 5 mg Cd/kg diet		
		Main effect of Cd	Main effect of AE	Main effect of Cd + AE	Main effect of Cd	Main effect of AE	Main effect of Cd + AE
3	GSH	-	-	-	-	-	-
	GSSG	-	-	-	NS	NS	15.09***
	GSH/GSSG	-	-	-	NS	25.60***	11.05**
	TSH	NS	6.444*	12.93**	7.320*	NS	47.82*
10	GSH	13.83***	NS	NS	NS	NS	7.440*
	GSSG	6.901*	8.813*	4.312*	27.43***	10.04**	NS
	GSH/GSSG	NS	8.450*	9.319**	NS	4.852*	5.543*
	TSH	NS	5.346*	26.05*	NS	NS	19.47*
17	GSH	8.085*	NS	NS	10.95**	5.444*	NS
	GSSG	NS	10.98**	NS	NS	33.16***	13.64***
	GSH/GSSG	NS	15.59***	NS	NS	34.03***	15.52***
	TSH	5.267*	NS	14.45***	13.43**	NS	7.760**
24	GSH	NS	NS	8.384*	NS	4.752*	10.68**
	GSSG	10.887**	44.01***	NS	11.22**	81.91***	NS
	GSH/GSSG	6.022*	24.41***	NS	NS	41.89***	NS
	TSH	NS	30.21***	NS	NS	35.90***	NS

[1] The rats received the 0.1% aqueous AE and Cd in diet at the concentration of 1 or 5 mg/kg for 3–24 months.

[2] The results of the ANOVA/MANOVA analysis are presented as *F* values and the level of statistical significance (*p*). *F* values having $p < 0.05$ were considered statistically significant (* $p < 0.05$, ** $p < 0.01$, *** $p < 0.001$). NS – not statistically significant ($p > 0.05$). In the case when a one way-analysis of variance (ANOVA, Duncan's multiple range test) revealed any influence of the co-administration of Cd and AE on the investigated parameter, a two-way analysis of variance (ANOVA/MANOVA, test *F*) was conducted in aim to discern possible interactive and independent impact of Cd and AE on this parameter.

Table S7. Effect of the extract from the berries of *Aronia melanocarpa* L. (AE) on the concentration of thioredoxin (Trx) in the liver of rats exposed to cadmium (Cd) [1,2].

Group	Duration of the Experiment			
	**3 Months**	**10 Months**	**17 Months**	**24 Months**
**Trx (ng/mg protein)**				
Control	0.152 ± 0.015	0.236 ± 0.017	0.152 ± 0.009	0.153 ± 0.016
AE	0.146 ± 0.018	0.239 ± 0.017	0.158 ± 0.015	0.186 ± 0.026
Cd_1	0.163 ± 0.016	0.258 ± 0.007	0.174 ± 0.016	0.173 ± 0.021
Cd_1 + AE	0.138 ± 0.008	0.246 ± 0.019	0.143 ± 0.019	0.199 ± 0.014
Cd_5	0.150 ± 0.017	0.196 ± 0.011	0.168 ± 0.012	0.167 ± 0.007
Cd_5 + AE	0.155 ± 0.017	0.235 ± 0.017	0.162 ± 0.011	0.243 ± 0.016$^{** \dagger\dagger \ddagger}$

[1] The rats received the 0.1% aqueous AE and Cd in diet at the concentration of 1 or 5 mg/kg for 3–24 months.

[2] Data are represented as mean ± SE for 8 rats (except for 7 animals in the AE, Cd_1 and Cd_5 groups after 24 months). Statistically significant differences (ANOVA, Duncan's multiple range test) compared to the control group (** $p < 0.01$), group intoxicated with 5 mg Cd/kg diet alone (†† $p < 0.01$), and group receiving AE alone (‡ $p < 0.05$) are marked.

Table S8. Main and interactive effects of cadmium (Cd) and the extract from the berries of *Aronia melanocarpa* L. (AE) on the concentrations of hydrogen peroxide (H_2O_2), myeloperoxidase (MPO), and xanthine oxidase (XOD) in the liver of rats [1,2].

Duration (months)	Parameter	Exposure to 1 mg Cd/kg diet			Exposure to 5 mg Cd/kg diet		
		**Main effect of Cd**	**Main effect of AE**	**Main effect of Cd + AE**	**Main effect of Cd**	**Main effect of AE**	**Main effect of Cd + AE**
**3**	**H_2O_2**	NS	13.93^{***}	NS	NS	9.818^{**}	NS
	**MPO**	NS	NS	NS	NS	NS	6.790^{*}
	**XOD**	NS	13.35^{**}	NS	NS	15.45^{***}	NS
**10**	**H_2O_2**	18.70^{***}	26.93^{***}	21.02^{***}	26.81^{***}	27.75^{***}	21.05^{***}
	**MPO**	13.01^{**}	7.045^{*}	10.97^{**}	NS	NS	5.710^{*}
	**XOD**	14.09^{***}	5.003^{*}	11.72^{***}	NS	NS	12.75^{**}
**17**	**H_2O_2**	29.54^{***}	57.73^{***}	78.96^{***}	61.98^{***}	103.3^{***}	137.6^{***}
	**MPO**	4.684^{*}	22.84^{***}	8.230^{**}	6.180^{*}	22.83^{***}	8.717^{**}
	**XOD**	NS	30.02^{***}	11.76^{**}	15.13^{***}	58.18^{***}	36.78^{***}
**24**	**H_2O_2**	70.28^{***}	71.69^{***}	63.92^{***}	60.62^{***}	67.56^{***}	60.72^{***}
	**MPO**	NS	7.622^{*}	5.008^{*}	NS	13.00^{**}	7.976^{**}
	**XOD**	6.310^{*}	13.15^{**}	20.72^{***}	9.778^{**}	23.71^{***}	32.74^{***}

[1] The rats received the 0.1% aqueous AE and Cd in diet at the concentration of 1 or 5 mg/kg for 3–24 months.

[2] The results of the ANOVA/MANOVA analysis are presented as *F* values and the level of statistical significance (*p*). *F* values having $p < 0.05$ were considered statistically significant (* $p < 0.05$, ** $p < 0.01$, *** $p < 0.001$). NS – not statistically significant ($p > 0.05$). In the case when a one way-analysis of variance (ANOVA, Duncan's multiple range test) revealed any influence of the co-administration of Cd and AE on the investigated parameter, a two-way analysis of variance (ANOVA/MANOVA, test *F*) was conducted in aim to discern possible interactive and independent impact of Cd and AE on this parameter.

Table S9. Main and interactive effects of cadmium (Cd) and the extract from the berries of *Aronia melanocarpa* L. (AE) on the total antioxidative status (TAS), total oxidative status (TOS), index of oxidative stress (OSI), and the concentration of malondialdehyde (MDA) in the liver of rats [1,2].

Duration (months)	Parameter	Exposure to 1 mg Cd/kg diet			Exposure to 5 mg Cd/kg diet		
		Main effect of Cd	Main effect of AE	Main effect of Cd + AE	Main effect of Cd	Main effect of AE	Main effect of Cd + AE
3	TOS	-	-	-	-	-	-
	TAS	-	-	-	-	-	-
	OSI	-	-	-	NS	9.160**	6.344*
	MDA	11.864**	NS	4.271*	11.08**	NS	4.455*
10	TOS	27.04***	NS	NS	7.025*	NS	NS
	TAS	4.410*	6.670*	6.062*	NS	NS	NS
	OSI	44.87***	101.2***	50.51***	11.05**	17.93**	5.934*
	MDA	10.102**	NS	NS	17.86***	NS	13.79***
17	TOS	26.68***	NS	NS	-	-	-
	TAS	NS	NS	NS	NS	NS	NS
	OSI	22.54***	21.73***	31.99***	13.85***	13.60**	23.47***
	MDA	8.175**	4.691*	NS	15.15***	NS	NS
24	TOS	-	-	-	44.22***	6.251*	NS
	TAS	NS	6.094*	14.79***	13.85***	13.60**	23.47***
	OSI	10.85**	NS	11.87**	21.13***	31.37***	54.51***
	MDA	NS	NS	NS	6.610*	4.519*	NS

[1] The rats received the 0.1% aqueous AE and Cd in diet at the concentration of 1 or 5 mg/kg for 3–24 months.

[2] The results of the ANOVA/MANOVA analysis are presented as *F* values and the level of statistical significance (*p*). *F* values having $p < 0.05$ were considered statistically significant (* $p < 0.05$, ** $p < 0.01$, *** $p < 0.001$). NS – not statistically significant ($p > 0.05$). In the case when a one way-analysis of variance (ANOVA, Duncan's multiple range test) revealed any influence of the co-administration of Cd and AE on the investigated parameter, a two-way analysis of variance (ANOVA/MANOVA, test *F*) was conducted in aim to discern possible interactive and independent impact of Cd and AE on this parameter.

Table S10. The effect of the extract from the berries of *Aronia melanocarpa* L. (AE) on the absolute and the relative weight of the liver of rats exposed to cadmium (Cd) [1,2].

Group	Duration of the Experiment			
	**3 Months**	**10 Months**	**17 Months**	**24 Months**
	**Absolute Weight of the Liver (g)**			
Control	8.0872 ± 0.8266	8.5750 ± 0.8857	10.798 ± 1.617339	12.328 ± 1.8483
AE	8.2253 ± 0.8418	9.8812 ± 1.8749	11.443 ± 1.624754	11.560 ± 2.0207
Cd_1	7.7314 ± 0.3937	9.0650 ± 0.5659	10.670 ± 2.220122	13.154 ± 1.7681
Cd_1 + AE	8.1927 ± 0.3578	9.1725 ± 1.3814	10.685 ± 1.446227	13.046 ± 1.5232
Cd_5	8.3599 ± 0.7913	11.165 ± 1.9868$^{**\ \#\#}$	10.033 ± 1.558259	14.402 ± 0.8830$^{*\ \ddagger\ddagger}$
Cd_5 + AE	7.9034 ± 1.2700	9.0675 ± 0.9645††	10.035 ± 1.676024	11.238 ± 1.8873††
	**Relative Weight of the Liver (g/100 g b.w.)**			
Control	2.5547 ± 0.1551	2.0023 ± 0.1900	2.0440 ± 0.1498	2.0911 ± 0.1560
AE	2.6033 ± 0.1859	2.2337 ± 0.3037	2.2265 ± 0.2835	2.0300 ± 0.2595
Cd_1	2.5243 ± 0.1106	2.0620 ± 0.1581	2.1013 ± 0.1899	2.2094 ± 0.2086
Cd_1 + AE	2.7272 ± 0.2111†	2.1240 ± 0.3727	2.1133 ± 0.1716	2.2726 ± 0.2224
Cd_5	2.6567 ± 0.1445	2.5101 ± 0.3535$^{**\ \#\#}$	1.9926 ± 0.1395‡	2.5357 ± 0.2523$^{***\ \ddagger\ddagger\ddagger\ \#}$
Cd_5 + AE	2.4807 ± 0.2392$^{\#}$	2.1400 ± 0.1078†	1.9873 ± 0.2358‡	2.0022 ± 0.2224$^{\dagger\dagger\dagger\ \#}$

[1] The rats received the 0.1% aqueous AE and Cd in diet at the concentration of 1 or 5 mg/kg for 3–24 months.

[2] Data are represented as mean ± SE for 8 rats (except for 7 animals in the AE, Cd_1, and Cd_5 groups after 24 months). Statistically significant differences (ANOVA, Duncan's multiple range test): * $p < 0.05$, ** $p < 0.01$, *** $p < 0.001$ vs. control group; † $p < 0.05$, †† $p < 0.01$, ††† $p < 0.001$ vs. respective group intoxicated with Cd alone; ‡ $p < 0.05$, ‡‡ $p < 0.01$, ‡‡‡ $p < 0.001$ vs. group receiving AE alone; $^{\#}$ $p < 0.05$, $^{\#\#}$ $p < 0.01$ vs. respective group receiving the 1 mg Cd/kg diet (alone or with AE).

Table S11. Main and interactive effects of cadmium (Cd) and the extract from the berries of *Aronia melanocarpa* L. (AE) on the activities of alanine aminotransferase (ALT) and aspartate transaminase (AST) in the serum of rats [1,2].

Duration (months)	Parameter	Exposure to 1 mg Cd/kg diet			Exposure to 5 mg Cd/kg diet		
		Main effect of Cd	Main effect of AE	Main effect of Cd + AE	Main effect of Cd	Main effect of AE	Main effect of Cd + AE
**3**	**ALT**	-	-	-	-	-	-
	**AST**	-	-	-	5.788*	NS	7.686**
**10**	**ALT**	25.25***	NS	NS	15.81***	28.52***	4.275*
	**AST**	7.127*	NS	NS	11.46**	NS	NS
**17**	**ALT**	NS	13.38**	14.46***	NS	11.32**	12.40**
	**AST**	27.01***	NS	NS	6.171*	NS	NS
**24**	**ALT**	NS	NS	6.342*	NS	NS	12.97**
	**AST**	NS	NS	NS	10.10**	NS	NS

[1] The rats received the 0.1% aqueous AE and Cd in diet at the concentration of 1 or 5 mg/kg for 3–24 months.

[2] The results of the ANOVA/MANOVA analysis are presented as *F* values and the level of statistical significance (*p*). *F* values having $p < 0.05$ were considered statistically significant (* $p < 0.05$, ** $p < 0.01$, *** $p < 0.001$). NS – not statistically significant ($p > 0.05$). In the case when a one way-analysis of variance (ANOVA, Duncan's multiple range test) revealed any influence of the co-administration of Cd and AE on the investigated parameter, a two-way analysis of variance (ANOVA/MANOVA, test *F*) was conducted in aim to discern possible interactive and independent impact of Cd and AE on this parameter.

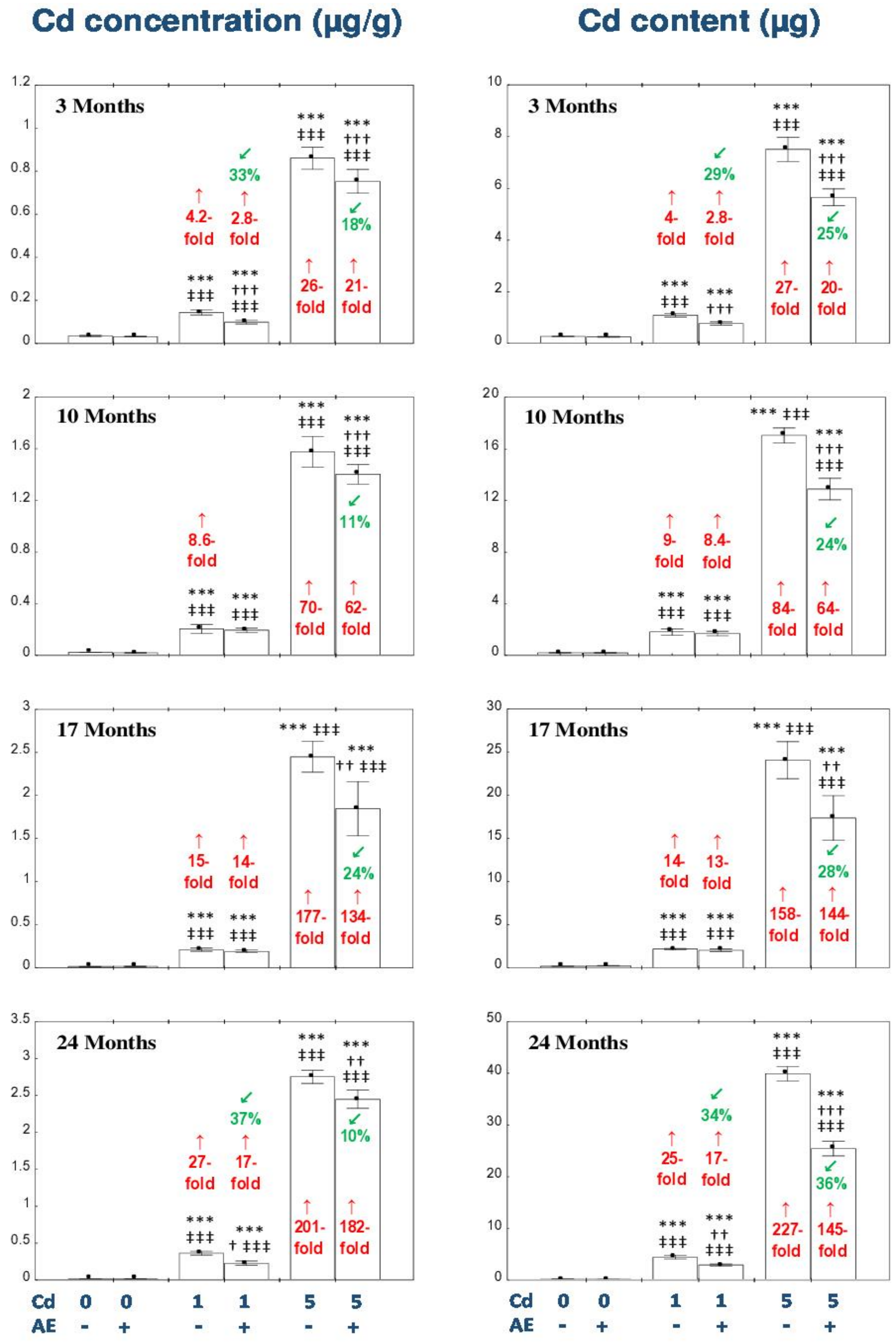

Figure S1. The effect of the extract from the berries of *Aronia melanocarpa* L. (AE) on the concentration and content of cadmium (Cd) in the liver of rats exposed to this metal. The rats received Cd in the diet at the concentration of 0, 1, and 5 mg Cd/kg and/or 0.1% aqueous AE (+) or not (-). Data are presented as mean ± SE for 8 rats, except for 7 animals in the AE, Cd_1, and Cd_5 group after 24 months. Statistically significant differences (ANOVA, Duncan's multiple range test): $^{***}p < 0.001$ vs. control group; $^{††}p < 0.01$, $^{†††}p < 0.001$ vs. respective group intoxicated with Cd alone; $^{‡‡‡}p < 0.001$ vs. group receiving AE alone. Numerical values in bars or above the bars disclose the percentage changes or factors of changes in comparison to the control group (↑, increase) or the respective group receiving Cd alone (↙, decrease). Detailed data on Cd concentration in the liver are presented in Table S1 [11]. Cd content in the liver in the control group reached 0.27510 ± 0.0265 µg, 0.2024 ± 0.0129 µg, 0.1525 ± 0.0216 µg, and 0.1747 ± 0.0231 µg after 3, 10, 17, and 24 months, respectively [11].

References

1. Mężyńska, M.; Brzóska, M.M. Review of polyphenol-rich products as potential protective and therapeutic factors against cadmium hepatotoxicity. *J. Appl. Toxicol.* **2018**, *39*, 117–145, doi:10.1002/jat.3709.
2. Cuypers, A.; Plusquin, M.; Remans, T.; Jozefczak, M.; Keunen, E.; Gielen, H.; Opdenakker, K.; Nair, A.R.; Munters, E.; Artois, T.J.; Nawrot, T.; Vangronsveld, J.; Smeets, K. Cadmium stress: an oxidative challenge. *Biometals* **2010**, *23*, 927–940, doi:10.1007/s10534-010-9329-x.
3. Klebanoff, S.J. Myeloperoxidase. *Proc. Assoc. Am. Physicians* **1999**. *111*, 383–389, doi:10.1111/paa.1999.111.5.383
4. Chung, H.Y.; Baek, B.S.; Song, S.H.; Kim, M.S.; Huh, J.I.; Shim, K.H.; Kim, K.W.; Lee, K.H. Xanthine dehydrogenase/xanthine oxidase and oxidative stress. *Age (Omaha)* **1997**, *20*, 127–140, doi:10.1007/s11357-997-0012.
5. Gong, P.; Chenb, F.; Wang, L.; Wang, J.; Jina, S.; Ma, Y. Protective effects of blueberries (*Vaccinium corymbosum* L.) extract against cadmium-induced hepatotoxicity in mice. *Environ. Toxicol. Pharmacol.* **2014**, *37*, 1015–1027, doi:10.1016/j.etap.2014.03.017.
6. Brzóska, M.M.; Rogalska, J.; Roszczenko, A.; Gałażyn-Sidorczuk, M.; Tomczyk, M. The mechanism of the osteoprotective action of a polyphenol-rich *Aronia melanocarpa* extract during chronic exposure to cadmium is mediated by the oxidative defense system. *Planta Med.* **2016**, *82*, 621–631, doi:10.1055/s-0042-103593.
7. El-Boshy, M.E.; Risha, E.F.; Abdelhamid, F.M.; Mubarak, M.S.; Hadda, T.B. Protective effects of selenium against cadmium induced hematological disturbances, immunosuppressive, oxidative stress and hepatorenal damage in rats. *J. Trace. Elem. Med. Biol.* **2015**, *29*, 104–110, doi:10.1016/j.jtemb.2014.05.009.
8. Renugadevi, J.; Prabu, S.M. Cadmium-induced hepatotoxicity in rats and the protective effect of naringenin. *Exp. Toxicol. Pathol.* **2010**, *62*, 171–181, doi:10.1016/j.etp.2009.03.010.
9. Abarikwu, S.O.; Njoku, R.C.; Lawrence, C.J.; Charles, I.A.; Ikewuchi, J.C. Rutin ameliorates oxidative stress and preserves hepatic and renal functions following exposure to cadmium and ethanol. *Pharm. Biol.* **2017**, *55*, 2161–2169, doi:10.1080/13880209.2017.1387575.
10. Zhang, Y.; den Braver-Sewradj, S.P.; den Braver, M.W.; Hiemstra, S.; Vermeulen, N.P.E.; van de Water, B.; Commandeur, J.N.M.; Vos, J.C. Glutathione *S*-transferase P1 protects against amodiaquine quinoneimines-induced cytotoxicity but does not prevent activation of endoplasmic reticulum stress in HepG2 cells. *Front. Pharmacol.* **2018**, *9*, 388, doi:10.3389/fphar.2018.00388.
11. Brzóska, M.M.; Gałażyn-Sidorczuk, M.; Jurczuk, M.; Tomczyk, M. Protective effect of *Aronia melanocarpa* polyphenols on cadmium accumulation in the body: A study in a rat model of human exposure to this metal. *Curr. Drug Targets* **2015**, *16*, 1470–1487.
12. Brzóska, M.M.; Rogalska, J.; Gałażyn-Sidorczuk, M.; Jurczuk, M.; Roszczenko, A.; Tomczyk, M. Protective effect of *Aronia melanocarpa* polyphenols against cadmium-induced disorders in bone metabolism: A study in a rat model of lifetime human exposure to this heavy metal. *Chem. Biol. Interact.* **2015**, *229*, 132–146, doi:10.1016/j.cbi.2015.01.
